# Diversity of Lifeways in Early Antillean Societies: A Multi‐Isotope Approach

**DOI:** 10.1002/ajpa.70039

**Published:** 2025-04-16

**Authors:** Yadira Chinique de Armas, William Mark Buhay, Ulises Miguel González Herrera, Silvia Teresita Hernández Godoy, Jorge Fernando Garcell Domínguez, Luis Manuel Viera Sanfiel, José Armando Caraballo Yera, Mirjana Roksandic, Jason Laffoon

**Affiliations:** ^1^ University of Winnipeg Winnipeg Canada; ^2^ Instituto Cubano de Anthropología Havana Cuba; ^3^ Universidad de Matanzas Matanzas Cuba; ^4^ Grupo de Investigación y Desarrollo de la Dirección de Cultura de Matanzas Matanzas Cuba; ^5^ Comisión Nacional de Monumentos de Cuba Havana Cuba; ^6^ Parque Nacional Caguanes Sancti Spiritus Cuba; ^7^ Faculty of Archaeology Leiden University Leiden the Netherlands; ^8^ Faculty of Science Vrije Universiteit Amsterdam the Netherlands

**Keywords:** Caribbean Archaic, diversity of lifeways, paleodiet, paleomobility, stable isotopes

## Abstract

**Objectives:**

In this paper, we sought to examine whether people with different lifeways, as evidenced by their mobility patterns and dietary practices, inhabited the Antilles in early precolonial time. We also aimed to explore spatiotemporal trends.

**Materials and Methods:**

New and previously published enamel strontium, oxygen, and carbon isotope data were combined with bone apatite carbon and bone collagen carbon and nitrogen isotope data to assess the mobility and diet of 146 individuals from eight early precolonial sites from Cuba.

**Results:**

At least three patterns of mobility, associated with different dietary signals, were identified. In contrast with the low ^87^Sr/^86^Sr and *δ* ^13^C_en_ variability found in Canímar Abajo (CA) between bce 1320 and 807, more variability in dietary practices and higher mobility was apparent in later groups. Between bce 116 and 241 ce, individuals from Playa del Mango showed high mobility within the Cauto region, likely associated with food procurement between inland and coastal areas. From at least 174 ce, a moderate pattern of mobility and a diversity of dietary traditions could be observed among groups from western sites. At least three general dietary patterns were observed, ranging from a 100% C_3_ diet to 70:30 C_3_/C_4_ and, in the case of CA, a higher dependence on marine/C_4_ resources.

**Conclusions:**

The differences observed in both mobility and diet between and within populations support the notion that groups with different lifeways inhabited the Antilles in precolonial times. This diverse mosaic of cultural traits defies attempts to group them into broad categories for regional studies of biological and cultural traits.

## 1 Introduction

The ancient Caribbean was the seat of immense biocultural variation, manifested in diverse phenotypes, technological assemblages, subsistence practices, political formations, and, as discussed in the present work, varied lifeways. This diverse and dynamic view of the ancient Caribbean was limited by Irving Rouse's influential model of migrations and cultural dispersal (Rouse 1992), firmly embedded in the culture history paradigm, and widely questioned in current Caribbean archaeology (Keegan 1985, 1994, 2007; Keegan and Hoffman 2017, Rodríguez Ramos 2010, 2013; Rodríguez Ramos et al. 2013). Following Rousean systematics, the first settlers of the region are known as Lithic and Archaic Age groups, which he considered to be bands of highly mobile groups whose subsistence depended exclusively on the acquisition of food sources obtained through gathering, hunting, and fishing (Rouse 1972, 1992). Since the characterization of the Lithic Age focused on stone tool classifications, the biological and cultural traits of the human populations who produced such tools are not well understood (Rodríguez Ramos, González Herrera, and Chinique de Armas 2023). Consequently, most biological and cultural information about the earliest settlers of the insular Caribbean is based on studies of sites ascribed to the Archaic Age. Radiocarbon dates from the Greater Antilles indicate that these first human populations occupied the Antilles as early as the fifth millennium bce (Cooper 2010; Rodríguez Ramos, Rodríguez, and Pestle 2023; Roksandic et al. 2015), although skeletal remains are confirmed only as far back as the third millennium bce.

Recent paleoethnobotanical and isotopic studies have substantially modified traditional views of the lifeways and diversity of precolonial Antillean societies. Although starch analyses demonstrated that the production of domesticated plants and cultivars was a widespread phenomenon in the early horizon of the region (Chinique de Armas et al. 2015; Chinique de Armas, González Herrera, et al. 2022; Pagán and Mickleburgh 2022; Pagán et al. 2005; Rodríguez Suárez et al. 2020), isotopic data from Canímar Abajo in Cuba and Ortiz in Puerto Rico found less isotopic diversity than expected for groups assumed to have high residential mobility (Pestle et al. 2023; Chinique de Armas et al. 2025). These results support the notion that at least some populations in the Antilles were not mobile bands of fisher/hunter gatherers.

In contrast with the assumed biocultural homogeneity that underlies most regional studies involving early Antillean groups (e.g., Lalueza‐Fox et al. 2003), a high diversity of dietary traditions (Chinique de Armas et al. 2016; Chinique de Armas, González Herrera, et al. 2022), including breastfeeding and weaning trajectories (Chinique de Armas et al. 2017; Chinique de Armas, Mavridou, et al. 2022; Chinique de Armas and Pestle 2018), technological assemblages (Guarch 1990; Kozlowski 1975), burial practices (González Herrera et al. 2021), phenotypes (Bolufé 2015), and possibly genotypes (Nägele et al. 2020) has been identified among individuals from early sites in Cuba. The central position of the archipelago in the Caribbean and the extent of its territory likely facilitated the convergence of populations from different neighboring regions with diverse biological and cultural backgrounds, shaping the cultural mosaic present in the archaeological record (Guarch 1990).

In this paper, we sought to analyze enamel strontium (^87^Sr/^86^Sr), enamel oxygen (*δ* ^18^O_en_), and carbon (*δ* ^13^C_en_), bone apatite carbon (*δ* ^13^C_ap_), and bone collagen carbon (*δ* ^13^C_co_) and nitrogen (*δ* ^15^N_co_) isotope data from eight early sites from Cuba to examine the question of whether people with different lifeways, as evidenced by their mobility patterns and dietary practices, inhabited the Greater Antillean Islands in early precolonial times. We also sought to explore regional and chronological trends.

### 1.1 Early Sites in Cuba: Cultural Diversity and Ecological Settings

The early groups of Cuba have been known by different names, including Ciboney (Guayabo Blanco and Cayo Redondo types) (Boomert 2000; Cruxent and Rouse 1961; Keegan and Hoffman 2017), *Preagroalfareros* (Tabío 1984), *Apropiadores* (Guarch 1990), and *Pretribales* (Alonso et al. 2015). As a result of the use of historical materialism as a hermeneutic device, most classification systems reflected the interest of Cuban archaeologists in economic systems and techno‐typologies (Guarch 1990; Tabío 1984; Tabío and Rey 1979). Understanding of the diversity of early populations was limited by the traditional archaeological view of Cuban precolonial history as a linear evolutionary process from simple to complex cultural expressions, with groups isolated within specific territories (Ulloa 2013).

**FIGURE 1:**
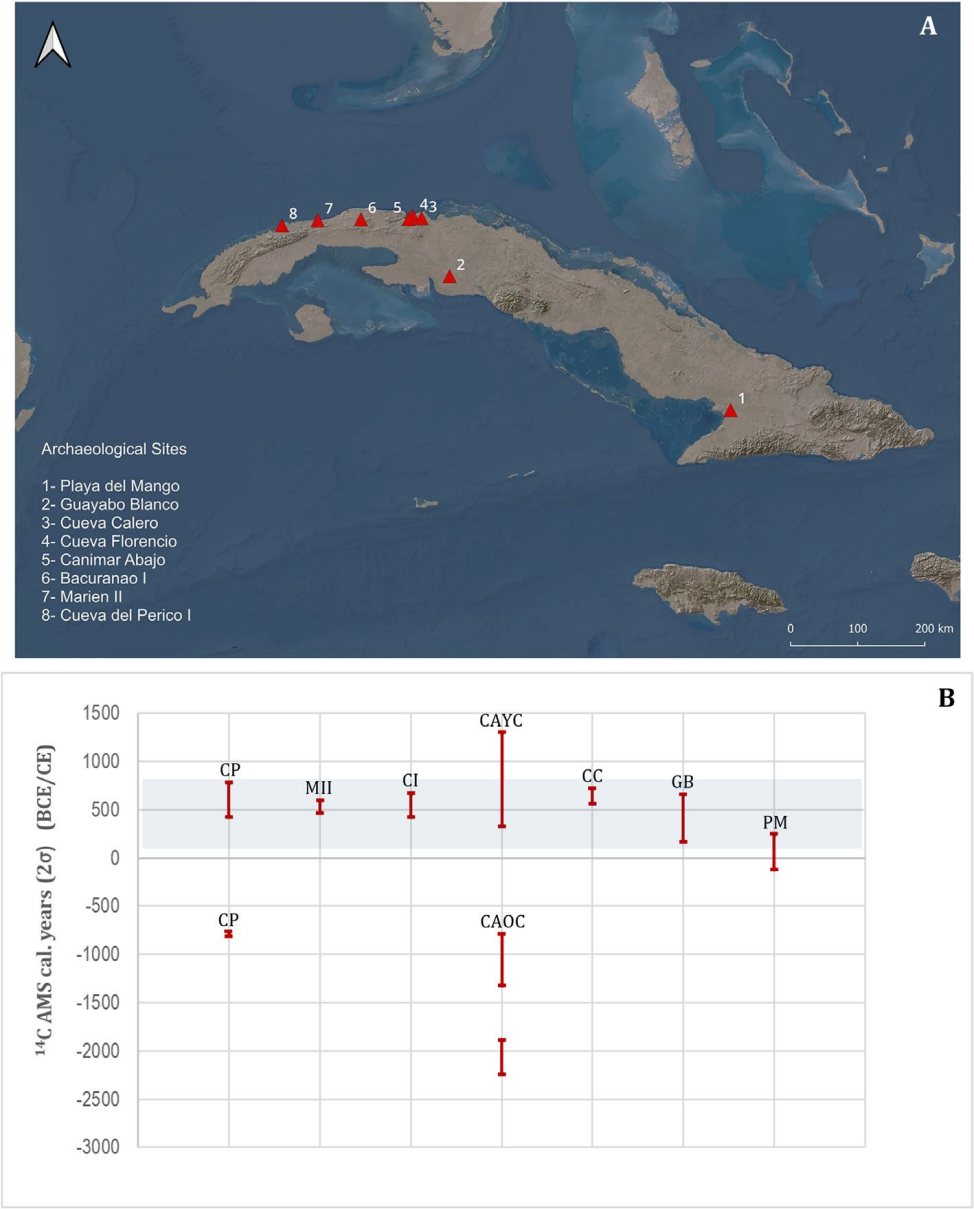
(A) Location of sites included in this study. (B) Available AMS radiocarbon dates from human remains of the sites included in this study. CP: Cueva del Perico; MII: Marien II; CI: Bacuranao I; CAYC: Canímar Abajo younger cemetery; CAOC: Canímar Abajo older cemetery; CC: Cueva Calero; GB: Guayabo Blanco; PM: Playa del Mango.

The sites included in this paper were selected based on the availability of human remains and to represent the biocultural and geographic diversity of early Cuban groups and the ecosystems that they occupied (Supporting Information: Appendix 1). The Banwaroid tradition, or Ciboney Cayo Redondo, is represented by Playa del Mango (cal. bce 116–241 ce [2*σ*]) in the Río Cauto Valley, Granma (20° 33′ 14″ N, 76° 59′ 9″ W) (Chinique de Armas et al. 2020). The Manicuaroid tradition, or Ciboney Guayabo Blanco, is represented by the Guayabo Blanco site (cal. bce 174–657 ce [2*σ*]) in Ciénaga de Zapata, Matanzas, the biggest wetland in Cuba (22° 18′ 3″ N, 80° 55′ 23″ W) (Cosculluela 1965) (Figure 1A).

The sites of Cueva Florencio (dates are not available), Cueva Calero (cal. 566–715 ce [2*σ*]), and Canímar Abajo (older cemetery [OC] cal. bce 2237–790; younger cemetery [YC]: 332–1291 ce [2*σ*]) are on the north coast of Matanzas. Cueva Florencio is a coastal site in the vicinity of Carbonera (23° 4′50″ N, 81° 26′24″ W). The site was classified to the Ciboney Guayabo Blanco tradition. In contrast, Cueva Calero is an inland funerary site (23° 3′ 0′′ N, 81° 19′ 2′′ W) (Martínez and Rives 1990). In addition, we included the site of Canímar Abajo (23° 02′ 16″ N, 81° 29′ 47″ W) where the earliest human remains from Cuba have been excavated (Chinique de Armas et al. 2025, submitted) (Figure 1A).

Toward the west, three cave sites were included: Bacuranao I (or Cueva del Infierno) (cal. 426–666 ce [2*σ*]), in the municipality of San José, in Mayabeque (23° 2′9″ N, 82° 10′3″ W); Marien II (474–597 ce [2*σ*]) (82° 46′ 28″ W, 23° 1′ 19″ N), classified as Ciboney Cayo Redondo type (La Rosa and Robaina 1995); and Cueva del Perico I (bce 796–775 ce) in Artemisa (83° 16′ 29.87″ W, 22° 57′ 29.43″ N) (Pino and Alonso 1973) (Figure 1A). The sites selected have burials that are dated between bce 2237 and 1291 ce (Figure 1B).

### 1.2 The Use of Stable Isotopes to Study Paleomobility and Paleodiets in the Antilles

The insular Caribbean comprises continental and oceanic islands underlain by various combinations of igneous, metamorphic, and sedimentary bedrock (Dengo and Case 1990; Donovan and Jackson 1994). Strontium isotope variation is predominantly controlled by geolithology, where bedrock geology is generally the primary source of strontium for terrestrial ecosystems (Bentley 2006). In the case of the Caribbean, local bioavailable ^87^Sr/^86^Sr values vary according to two major sources of strontium: bedrock, which is locally variable, and marine‐derived strontium (sea‐spray and precipitation) (Laffoon et al. 2012) with a uniform ^87^Sr/^86^Sr of ~0.7092 (Bataille et al. 2012). Large‐scale data sets of baseline ^87^Sr/^86^Sr values derived from modern plants, modern and archaeological animal remains, and soil and water samples have demonstrated that the overall region possesses bioavailable ^87^Sr/^86^Sr ranging from ~0.7055 to 0.7095. Exceptions to this pattern include portions of the continental islands of Trinidad and Tobago, which have slightly higher ratios (Laffoon et al. 2012). At the insular scale, the Greater Antilles have bioavailable ^87^Sr/^86^Sr ranges that nearly encompass the entire Antillean range (Bataille et al. 2012; Giovas et al. 2016; Laffoon et al. 2012; Pestle 2013; Schulting et al. 2018).

This spatial patterning of bioavailable ^87^Sr/^86^Sr in the Caribbean offers both benefits and drawbacks for the application of strontium isotope analysis to investigate human mobility patterns. The relatively narrow local ^87^Sr/^86^Sr ranges for most sites make it relatively easy to identify non‐local outliers (immigrants), whereas the high degree of overlap in bioavailable ^87^Sr/^86^Sr between and within many islands makes it challenging to ascertain the origins of individuals migrating within the Antilles (Laffoon et al. 2012). The restricted macro‐regional range of bioavailable ^87^Sr/^86^Sr permits the identification of nonlocal migrants originating from continental origins outside of the Caribbean (e.g., Laffoon et al. 2017; Laffoon et al. 2018; Price et al. 2020; Schroeder et al. 2009).

Oxygen isotopes are also potentially useful paleomobility proxies in many regions (Lee‐Thorp 2008; White et al. 1998). Oxygen isotopes in human tissues such as enamel (*δ* ^18^O_en_) primarily reflect variation in consumed water sources, which is dominated by local precipitation in most archaeological contexts. In turn, *δ* ^18^O_en_ in precipitation is predominantly influenced by geographic (latitude, altitude, distance from coast) and climatic (precipitation, temperature) conditions (Ehleringer et al. 2008; Longinelli 1984; Luz et al. 1984). The main limitation of this approach is the wide range of other factors that can influence skeletal *δ* ^18^O values, including seasonality, breastfeeding, and cooking (Brettell et al. 2012; Lightfoot and O'Connell 2016; Pellegrini et al. 2016). Moreover, the Caribbean has relatively limited variation in *δ* ^18^O values, making it a rather poor proxy for elucidating human movements within the Antilles (Laffoon 2013). Similar to strontium, oxygen isotopes are better suited for identifying long‐distance migrations from other regions to the Caribbean and tend to work best as a complementary proxy when combined with strontium isotopes and other independent lines of evidence (Laffoon et al. 2017).

Isotope analysis of carbon from enamel, bone collagen, and bone apatite (*δ* ^13^C_en_, *δ* ^13^C_co_, and *δ* ^13^C_ap_, respectively) and nitrogen from bone collagen (*δ* ^15^N_co_) has a long history in bioarchaeological research in the Caribbean region (Keegan and DeNiro 1988; Schoeninger et al. 1983; Stokes 1998; Pestle 2010; Buhay et al. 2013; Chinique de Armas et al. 2015; Chinique de Armas, González Herrera, et al. 2022; Chinique de Armas et al. 2016). Notable inter‐island and inter‐site variation has been observed within the major archipelagoes, likely owing to a combination of ecological and cultural factors (Chinique de Armas et al. 2016; Krigbaum et al. 2013; Laffoon et al. 2016; Laffoon et al. 2020; Norr 2002; Pestle 2010, 2013; Schulting et al. 2021; Stokes 1998). This spatial patterning therefore permits stable carbon and nitrogen isotope values—traditionally used solely for dietary studies—to be used as a supplementary proxy for human mobility studies in the Caribbean region (Chinique de Armas et al. 2025; Laffoon et al. 2016, 2020; Laffoon et al. 2013).

## 2 Materials and Methods

### 2.1 Human Samples

A total of 60 human dental enamel samples from 51 individuals were processed for ^87^Sr/^86^Sr, *δ* ^18^O_en_, and *δ* ^13^C_en_ isotope analysis to reconstruct mobility and general diet, including individuals from Playa del Mango (PM, *n* = 24), Bacuranao I (CI, *n* = 32), and Marien II (MII, *n* = 4) (Table 1). Eight individuals from PM and one from CI had more than one tooth available for sampling, allowing a longitudinal study of their mobility or diet. The *δ* ^18^O_en_ and *δ* ^13^C_en_ values of CI were previously published in a study on weaning practices (Chinique de Armas, Mavridou, et al. 2022); *δ* ^13^C_en_ values from PM were previously published as part of a paleodietary reconstruction (Chinique de Armas, González Herrera, et al. 2022).

**TABLE 1:** General information of the samples included in this study: Playa del Mango (PM), Guayabo Blanco (GB), Cueva Florencio (Fl), Cueva Calero (CC), Canímar Abajo (older cemetery: CAOC, younger cemetery: CAYC), Bacuranao I (CI), Marién II (MII), Cueva del Perico I (CP).

ID	Site	Context	Teeth/Bone	Age at crown formation	cal bce/ce (2*σ*)	Median prob.
PM2_E‐1	PM	B4/C‐5; C‐14; C‐8 0.10–0.25 m	C	Childhood	48–235 ce	120
PM2_E‐2	PM	B4/C‐5; C‐8 0.10–0.25 m	M1/M3 Femur	Infancy/Adolescence Adulthood	15–225 ce	101
PM2_E‐3	PM	B4/C‐11; C‐10 0.10–0.25 m	M1/M2 Femur	Infancy/Childhood Adulthood	bce 116–75 ce	−16
PM2_E‐4	PM	B4/C‐11; C‐4 0.10–0.25 m	Femur	Adulthood	bce 52–58 ce	−6
PM2_E‐6	PM	B4 0.15 m	m2/M1	Infancy	n/a	n/a
PM2_E‐7	PM	B6/C‐I; C‐VI 0.10–0.25 m	Femur	Adulthood	57–177 ce	106
PM2_E‐9	PM	B6/C–H; C‐K 0.10–0.20 m	M1/M3 Femur	Infancy/Adolescence Adulthood	bce 114–25 ce	−42
PM2_E‐10	PM	B6/C‐J; C‐III 0.10–0.25 m	M1 Femur	Infancy Adulthood	79–216 ce	131
PM2_E‐11	PM	B6 0.10–0.25 m	M1/M3 Femur	Infancy/Adolescence Adulthood	n/a	n/a
PM2_E‐12	PM	B6/C‐III; C‐G 0.10–0.25	Femur	Adulthood	15–91 ce	59
PM2_E‐13	PM	B6/C‐F; C‐VI 0.10–0.25	M2 Femur	Childhood Adulthood	127–241 ce	180
PM2_E‐14	PM	B6/C‐III 0.10–0.20 m	M2/M3 Femur	Childhood/Adolescence Adulthood	47–140 ce	96
PM2_E‐15	PM	B6/C‐J 0.10–0.20 m	M1/M3 Femur	Infancy/Adolescence Adulthood	bce 3–81 ce	37
PM1_M43	PM	B1 Level 1	M1	Infancy	n/a	n/a
PM1_M46	PM	B1 Level 1	Femur	Adulthood	n/a	n/a
PM1_R4	PM	B1/R4 Level 1	M3	Adolescence	n/a	n/a
PM1_C‐E5	PM	B1/E5 Level 1	M1	Infancy	n/a	n/a
PM1_C‐T4	PM	B1/T4 Level 2	M3	Adolescence	n/a	n/a
PM1_eT4	PM	B1/T4 Level 2	M1/PM1	Infancy/childhood	n/a	n/a
PM2_N1	PM	B4/C‐5, 0.25 m	M1	Infancy	n/a	n/a
GB F1560	GB	Capa b/Capa c 0.60 m	Femur	Adulthood	n/a	n/a
GB F‐1190	GB	Capa b/Capa c 0.60 m	Femur	Adulthood	n/a	n/a
GB F‐1189	GB	Capa b/Capa c 0.60 m	Femur	Adulthood	527–644 ce	564
GB F‐1192	GB	Capa b/Capa c 0.60 m	Femur	Adulthood	n/a	n/a
GB F‐1561	GB	Capa b/Capa c 0.60 m	Femur	Adulthood	532–657 ce	590
Fl_1	Fl	n/a	Femur	Adulthood	n/a	n/a
Fl_2	Fl	n/a	Femur	Adulthood	n/a	n/a
CC_E‐1 IV A	CC	Área 1/Unidad 1 0.79 m	Femur	Adulthood	n/a	n/a
CC_E‐1 IV B	CC	Área 1/Unidad 1 0.80 m	Femur	Adulthood	n/a	n/a
CC _E‐IV	CC	Área 1/Unidad 5/Escaque 2 0.20–0.30 m	Femur	Adulthood	n/a	n/a
CC_E‐ XIV UH II	CC	Área 2/Trinchera 2/Sección B 0.70 m	Femur	Adulthood	n/a	n/a
CC_ E‐5	CC	Área 2/Trinchera 2/Sección F 1.04 m	Femur	Adulthood	n/a	n/a
CC _E‐1	CC	Área 2/Trinchera 1/Sección D 0.68–0,84 m	Femur	Adulthood	n/a	n/a
CC_E‐17	CC	Área 2/Trinchera 2 y 3/Sección E 0.54 m	Femur	Adulthood	n/a	n/a
CA_E‐19a	CAOC	C‐118/119; 1.7–1.8 m	Femur	Adulthood	bce 1134–930	−1060
CA_E‐19b	CAOC OC	C‐118/119; 1.7–1.8 m	Femur	Adulthood	n/a	n/a
CA_E‐87	CAOC	C‐120/154; 1.5–1.7 m	Femur	Adulthood	bce 1132–982	−1071
CA_E‐2	CAYC	C‐118/119; 0.34 m	Femur	Infancy Adulthood	601–686 ce	652
CA_E‐4	CAYC	C‐96; 0.3 m	Femur	Adulthood	n/a	n/a
CA_E‐6	CAYC	C‐96/119; 0.50–0.55 m	Femur	Adulthood	n/a	n/a
CA_E‐7	CAYC	C‐153; 0.36 m	Femur	Adulthood	332–565 ce	448
CA_E‐10	CAYC	C‐119; 0.67 m	Femur	Adulthood	599–677 ce	645
CA_E‐68	CAYC	C‐120; 0.3–0.4 m	Femur	Adulthood	n/a	n/a
CA_E‐69	CAYC	C‐117; 0.38–0.5 m	Femur	Adulthood	n/a	n/a
CA_E‐70	CAYC	C‐122; 0.0–0.3 m	Femur	Adulthood	n/a	n/a
CA_E‐72	CAYC	C‐120/121; 0.52 m	Femur	Adulthood	407–601 ce	496
CA_E‐74	CAYC	C‐116; 0.2–0.3 m	Femur	Adulthood	n/a	n/a
CA_E‐75	CAYC	C‐117; 0.72 m	Femur	Infancy Adulthood	578–653 ce	619
CA_E‐77	CAYC	C‐116/117/93/94; 0.22–0.52 m	Femur	Childhood Adulthood	664–894 ce	789
CA_E‐80	CAYC	C‐98/21; 0.7 m	Femur	Adulthood	n/a	n/a
CA_E‐82	CAYC	C‐116; 0.65 m	Femur	Adulthood	1222–1291 ce	1263
CA E‐83	CAYC	C‐150/151; 0.53–0.59 m	Femur	Infancy Adulthood	564–775 ce	666
CA_E‐84	CAYC	C‐150; 0.65 m	Femur	Adulthood	n/a	n/a
CA_E‐90	CAYC	C‐150; 0.2–0.3 m	Femur	Adulthood	n/a	n/a
CA_E‐91	CAYC	C‐150; 0.3–0.4 m	Femur	Adulthood	n/a	n/a
CI_AdultoB	CI	C‐3, 1.00 m, saco 79	C	Childhood	n/a	n/a
CI_E‐1	CI	C‐3, 1.00 m, saco 79	dm1	Infancy	638–666 ce	650
CI_E‐1C	CI	C‐3, 1.00 m, saco 79	dc/M1	Infancy	n/a	n/a
CI_E‐1D	CI	C‐3, 1.00 m, saco 79	dm2	Infancy	n/a	n/a
CI_E‐6 A	CI	C‐3, 1.95–1.98 m, saco 168	dm2	Infancy	n/a	n/a
CI_E‐21B	CI	C‐4, 2.44–3.15 m, saco 69	dm2	Infancy	n/a	n/a
CI_E‐21C	CI	C‐4, 2.44–3.15 m, saco 69	dc	Infancy	n/a	n/a
CI_E‐21E	CI	C‐4, 2.44–3.15 m, saco 69	M1	Infancy	n/a	n/a
CI_E‐21F	CI	C‐4, 2.44–3.15 m, saco 69	M1	Infancy	n/a	n/a
CI_E‐28A	CI	C‐4, 1.68–2.28 m, saco 48	dm2	Infancy	n/a	n/a
CI_E‐29A	CI	A‐3, 3.0–3.7 m, saco 38	dc	Infancy	n/a	n/a
CI_E‐30A	CI	A‐3, 1.36–2.22 m, saco 36	dm2	Infancy	n/a	n/a
CI_E‐31A	CI	B‐4, 2.0–2.20 m, saco 54	dm1	Infancy	n/a	n/a
CI_E‐34A	CI	B‐5, 2.0–2.80 m, saco 28	dm1	Infancy	n/a	n/a
CI_E‐35A	CI	C‐6, C‐5, 1.60–1.80 m, saco 166	dm2	Infancy	n/a	n/a
CI_E‐36A	CI	B‐5, 1.80 m, saco 103	dm1	Infancy	n/a	n/a
CI_E‐39A	CI	C‐5, 1.18–1.48, saco 110	M1	Infancy	n/a	n/a
CI_E‐40A	CI	C‐5, 6, D‐5, 6, 1.04–1.73, saco 43	dm2	Infancy	n/a	n/a
CI_E‐49A	CI	A‐3, 4, B‐3, 3.0–3.79, saco 26	dm2	Infancy	n/a	n/a
CI_E‐51A	CI	C‐4, 1.8, saco 72	dm2	Infancy	n/a	n/a
CI_I22	CI	Isolated remains	M1	Infancy	n/a	n/a
CI_I23	CI	Isolated remains	M1	Infancy	n/a	n/a
CI_I25	CI	Isolated remains	M1	Infancy	n/a	n/a
CI_I26	CI	Isolated remains	M1	Infancy	n/a	n/a
CI_I27	CI	Isolated remains	M1	Infancy	n/a	n/a
CI_I28	CI	Isolated remains	M1	Infancy	n/a	n/a
CI_I29	CI	Isolated remains	M1	Infancy	n/a	n/a
CI_I31	CI	Isolated remains	M1	Infancy	n/a	n/a
CI_I41	CI	Isolated remains	M1	Infancy	n/a	n/a
CI_I40	CI	Isolated remains	M1	Infancy	n/a	n/a
CI_I43	CI	Isolated remains	M1	Infancy	n/a	n/a
MII_E‐1	MII	C‐3; 0.0–0.10 m	M1	Infancy	474–597 ce	541
MII_E‐2	MII	C‐3; 0.112 m	M1	Infancy	473–596 ce	540
MII_E‐10	MII	B3; 0.139–0.232 m	Femur	Adulthood	n/a	n/a
MII_E‐15	MII	B‐2/B3/C2/C3; 0.273–0.303 m	Femur	Adulthood	n/a	n/a
MII_E‐18	MII	C‐4; 0.148–0.17 m	M1	Infancy	n/a	n/a
MII_E‐21	MII	C‐4; 0.244–0.315 m	M1 Femur	Infancy Adulthood	n/a	n/a
CP_Adulto A #2524	CP	Trinchera 1/Sección 1 1.30–1.50 m	Femur	Adulthood	n/a	n/a
CP_E‐36	CP	Trinchera 1/Sección 1 1.10–1.20 m	Femur	Adulthood	n/a	n/a
CP_E‐6A	CP	Cala 1 1.37 m	Femur	Adulthood	n/a	n/a
CP_E‐60	CP	Salón A/Cala de prueba Superficie	Femur	Adulthood	n/a	n/a
CP_E‐39	CP	Trinchera 1/Sección 1 1.50–1.60 m	Femur	Adulthood	n/a	n/a
CP_E‐26B	CP	Trinchera 1/Sección 1 1.50–1.60 m	Femur	Adulthood	n/a	n/a
CP_E‐61	CP	Salón A/Cala de prueba Superficie	Femur	Adulthood	650–775 ce	726
CP_E‐28	CP	Trinchera 1/Sección 1 1.50–1.60 m	Femur	Adulthood	n/a	n/a

*Note:* Dates were taken from Roksandic et al. (2015), Nägele et al. (2020) and Chinique de Armas et al. (2020, 2025, submitted).

Abbreviations: C: canine, I: incisor, PM: premolar, M1, M2, M3: permanent first, second and third molars.

The age range of enamel formation differs between deciduous and permanent teeth and between different tooth types (Hillson 2005). Human tooth enamel is not subject to turnover once it is fully formed (Nanci 2013), retaining the isotopic signals of the period of crown formation. Most samples were taken from first permanent molars (*n* = 12 from PM; *n* = 4 from MII; *n* = 14 from CI) or deciduous dentition (*n* = 1 from PM; *n* = 16 from CI). Crown formation of these teeth occurs during infancy (before a year for deciduous dentition and between 0 and 3 years for the first permanent molars) (Hillson 2005). The period of childhood (3–7 years) is better represented by premolars (*n* = 1 from PM), second molars (*n* = 3 from PM), and canines (*n* = 1 from CI), while the third permanent molars (*n* = 7 from PM) represent the period of adolescence (Hillson 2005).

The ^87^Sr/^86^Sr isotope data from 26 individuals from Canímar Abajo (CA) (Chinique de Armas et al. 2025), 17 individuals from Cueva del Perico I (CP) (Laffoon and Chinique de Armas Forthcoming), and 4 individuals from Ortiz (Pestle et al. 2023) were incorporated into the analysis to understand diversity in mobility patterns and diet among early Indigenous populations in the Antilles (Table 2).

**TABLE 2:** ^87^Sr/^86^Sr values of teeth from Canímar Abajo (CAOC: Older cemetery; CAYC: Younger cemetery), Cueva del Perico I (CP), and Ortiz (Or) site individuals.

^87^Sr/^86^Sr	CAOC^a^	CAYC^a^	CP^b^	Or^c^
*N*	23 (13)	21 (13)	17	4 (4)
Average	0.70895	0.70869	0.70826	0.70793
SD	0.00027	0.00044	0.00080	0.00022
Max	0.70908	0.70917	0.70873	0.70816
Min	0.70794	0.70748	0.70705	0.70756
Average (locals)	0.70901	0.70876	0.70830	—
SD	0.00009	0.00034	0.0000	—
Max	0.70908	0.70917	0.708360	—
Min	0.70870	0.70807	0.708161	—

*Note: N*: Sample number (number of individuals).

^a^ Data were taken from Chinique de Armas et al. (2025).

^b^ Laffoon and Chinique de Armas (Forthcoming).

^c^ Pestle et al. (2023).

In addition, new *δ* ^13^C_ap_ samples from 59 adult individuals from 8 early sites were processed and combined with new and previously published *δ* ^13^C_co_ and *δ* ^15^N_co_ data (Chinique de Armas et al. 2015; Chinique de Armas, González Herrera, et al. 2022; Chinique de Armas et al. 2016) to better understand the variation in subsistence strategies of early Indigenous groups in Cuba (*n* = 13 from PM, *n* = 5 from Guayabo Blanco [GB], *n* = 2 from Cueva Florencio [Fl], *n* = 7 from Cueva Calero [CC], *n* = 3 from CA OC; *n* = 18 from CA YC, *n* = 3 from MII, and *n* =8 from CP) (Table 1). The total number of individuals included in the analysis was 146 (180 samples).

Only individuals with *δ* ^13^C_co_, *δ* ^13^C_ap_, and *δ* ^15^N_co_ data were included in this study. We calculated the carbon isotopic composition of the dietary protein source for each group using the formula proposed by Pestle et al. (2014): *δ* ^13^C_protein_ (‰) = (0.78 × *δ* ^13^C_co_) − (0.58 × Δ^13^C_ap‐co_) − 4.7 (*r* ^2^ = 0.86). The formulas for estimating *δ* ^13^C from energy sources (*δ* ^13^C_energy_) and whole diet (*δ* ^13^C_diet_) were taken from Kellner and Schoeninger (2007): *δ* ^13^C_energy_ (‰) = (1.1 × *δ* ^13^C_ap_) − 8.4 (*r* ^2^ = 0.59); *δ* ^13^C_diet_ (‰) = (1.04 × *δ* ^13^C_ap_) − 9.2 (*r* ^2^ = 0.97). The *δ* ^13^C_co_ and *δ* ^13^C_ap_ were plotted against the model of Froehle et al. (2010). The *δ* ^13^C_co_, *δ* ^13^C_ap_, and *δ* ^15^N_co_ values were combined using the multivariate model proposed by Froehle et al. (2012). The discriminant function scores (Carbon: F1 = [0.322 × *δ* ^13^C_ap_] + [0.727 × *δ* ^13^C_co_] + [0.219 × *δ* ^15^N_co_] + 9.354; Nitrogen: F2 = [−0.393 × *δ* ^13^C_ap_] + [0.133 × *δ* ^13^C_co_] + [0.622 × *δ* ^15^N_co_] − 8.703) were calculated for all individuals and plotted against the model. The data that supports the findings of this study are available in the main text, and Supporting Information, of this article. All human remains included in this study were treated with dignity and respect.

### 2.2 Spatial Variation of ^87^Sr/ ^86^Sr and *δ* ^18^O_en_

**FIGURE 2:**
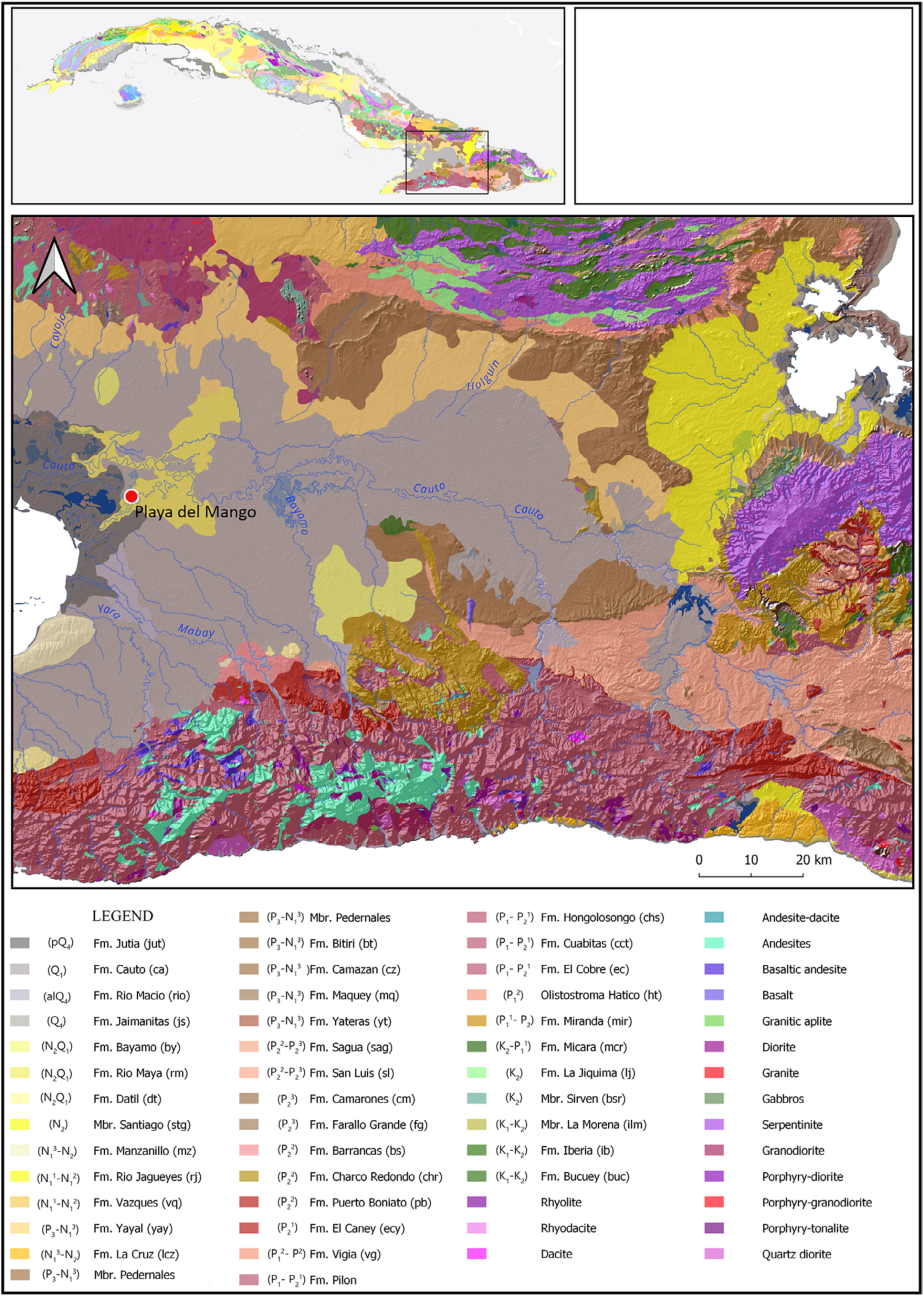
Geological map of Cauto Valley region. Fm: Formation; Q: Quaternary; N: Neogene; P: Paleogene; K: Cretaceous; J: Jurassic.

**FIGURE 3:**
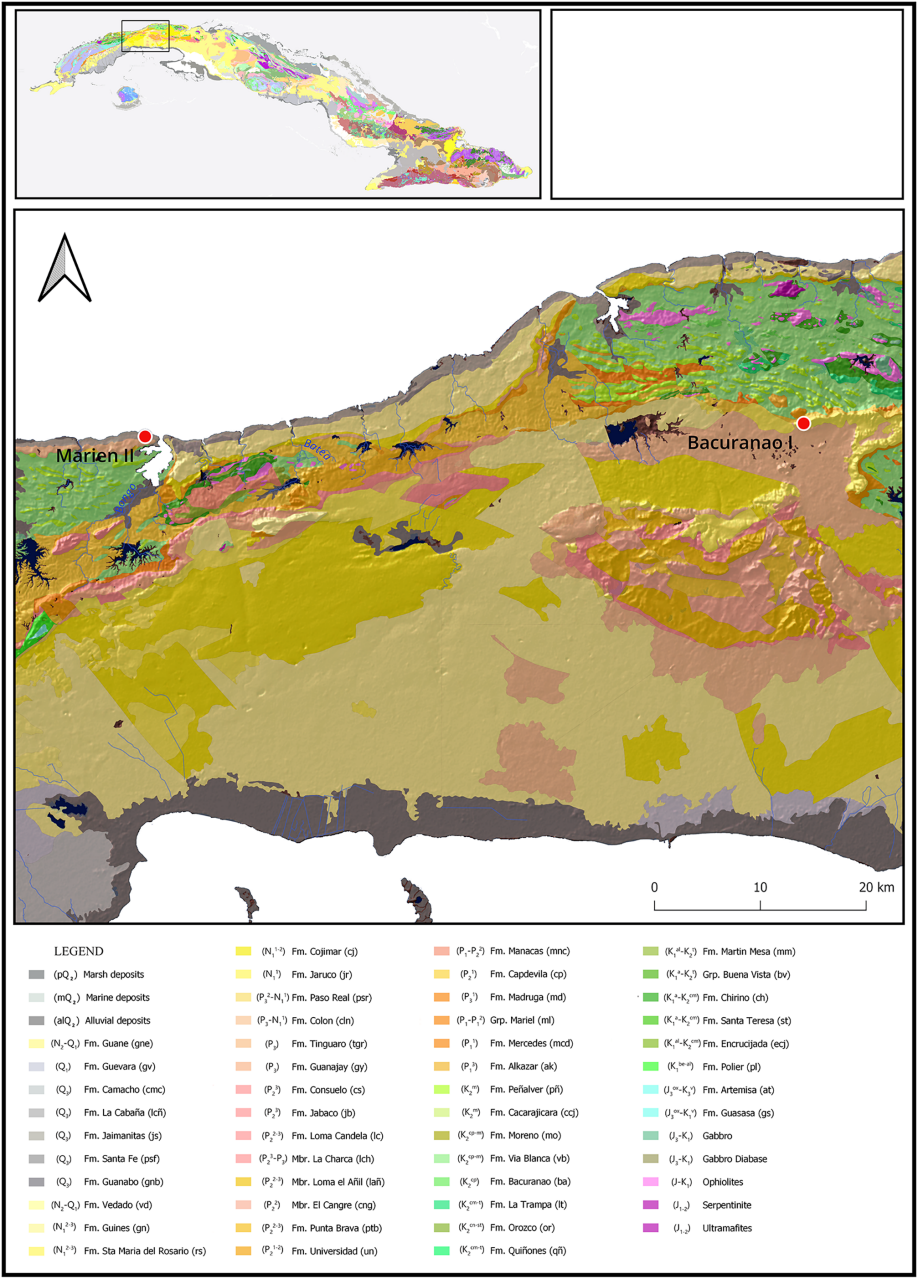
Geological map of west Cuba. Fm: Formation; Q: Quaternary; N: Neogene; P: Paleogene; K: Cretaceous; J: Jurassic.

The geological formations underlying the PM, CI, and MII sites are mostly composed of calcareous marls, limestones, conglomerates, and siliclastic limestones that are Pliocene‐Quaternary in age (Figures 2 and 3), with ^87^Sr/^86^Sr ratios expected to be 0.70913 ± 0.00038 (Laffoon et al. 2012). Values are expected to be lower at PM since the site is proximal to older carbonate sediments from the Eocene‐Miocene with expected values of 0.70846 ± 0.00093. For PM, we estimated the bioavailable ^87^Sr/^86^Sr and *δ* ^18^O_en_ of the site by studying tooth enamel of three archaeological hutias, rodent‐like mammals from the Antilles. Fauna was not available for the MII or CI sites.

Reported bioavailable and bedrock ^87^Sr/^86^Sr values from the broader region were taken into consideration to assess mobility and provenance (Laffoon et al. 2012). Values from archaeological samples were preferred over modern ones to avoid inaccuracies related to recent inputs of pollutants and fertilizers (Kamenov et al. 2009). For *δ* ^18^O variation, previously published enamel *δ* ^18^O data (Laffoon et al. 2013) and maps of Caribbean *δ* ^18^O isotope variation (Laffoon et al. 2017) were considered.

Individuals were considered nonlocals when the ^87^Sr/^86^Sr values were outside of the geoavailable ^87^Sr/^86^Sr range (seawater–bedrock) and their values were at least 3.0 apart from the mean standard deviation (*Z*‐scores ≤ −3.0 or ≥ 3.0) of the locals. In the case of PM, we also considered the bioavailable range established for the site.

### 2.3 Laboratory Procedure

All samples were prepared at the Faculty of Archaeology (Leiden University) and measured at the Faculty of Science (Vrije Universiteit Amsterdam). For teeth, dental crowns were mechanically cleaned by removing the outer surface with a diamond‐tipped drill bit attached to a rotary drill (Budd et al. 2000). For bones, small bone fragments were crushed into bone powder using a ceramic mortar and pestle that was cleaned between samples to prevent cross‐contamination. Sample processing for both enamel and bone bioapatite included a chemical pretreatment procedure (Bocherens et al. 2011) with a wash in 2.5% bleach (NaOCl), followed by a thorough rinsing in MilliQ water, proceeded by brief leaching in calcium acetate–buffered acetic acid (CH_3_COOH, pH = 4.75), and a final round of rinsing. Carbon (*δ* ^13^C_en_) and oxygen (*δ* ^18^O_en_) isotopes were measured on a Finnigan DeltaPlus isotope ratio mass spectrometer, interfaced with a Gasbench II. Long‐term reproducibility of the international reference material (NBS19) is ±0.1‰ (1*σ*) for *δ* ^13^C_en_ and ±0.2‰ (1*σ*) for *δ* ^18^O_en_. Stable isotope results are reported in the *δ* notation, in parts per 1000 (‰) relative to the international VPDB (carbon) and VSMOW (oxygen) standards.

For analysis of ^87^Sr/^86^Sr, 2–3 mg of powdered enamel was dissolved in 3 M nitric acid (HNO_3_). The strontium fraction was separated from the matrix via ion‐exchange column chromatography using Sr‐spec resin (Eichrom). Strontium isotope ratios were analyzed on a Thermo Scientific Triton Series multi‐collector thermal ion mass spectrometer. Ratios were corrected for mass fractionation using an exponential law and an ^86^Sr/^88^Sr ratio of 0.1194. Analysis of the international standard NBS987 produced a long‐term average ^87^Sr/^86^Sr ratio of 0.71024 ± 0.00004 (2*σ*) and total procedural blanks (< 100 pg Sr) were negligible relative to the Sr. contents of the samples.

### 2.4 Statistics

To detect outliers, the human ^87^Sr/^86^Sr values were tested using *Z*‐scores. Any deviation from the mean higher than 3.0 or lower than −3.0 was considered an outlier. The *δ* ^13^C_diet_, *δ* ^13^C_energy_, and *δ* ^13^C_protein_ mean comparisons between populations were performed in variables with at least three samples. Normal distribution was tested using the Shapiro–Wilks test. While *δ* ^13^C_diet_ (*W* = 0.97, *p* = 0.26) and *δ* ^13^C_energy_ (*W* = 0.97, *p* = 0.26) values followed a normal distribution, *δ* ^13^C_protein_ was not normally distributed (*W* = 0.96, *p* = 0.04). When the samples were normally distributed, comparisons between groups of samples were performed using a one‐way ANOVA (*F*) with a Tukey–Kramer post hoc test. For comparisons of more than two groups that did not fit the normal distribution, we used a Kruskal–Wallis (*H*) test with a Dunn test. Statistical significance was set at *α* = 0.05.

## 3 Results

### 3.1 Paleomobility and General Diet

#### 3.1.1 Playa del Mango Site

**FIGURE 4:**
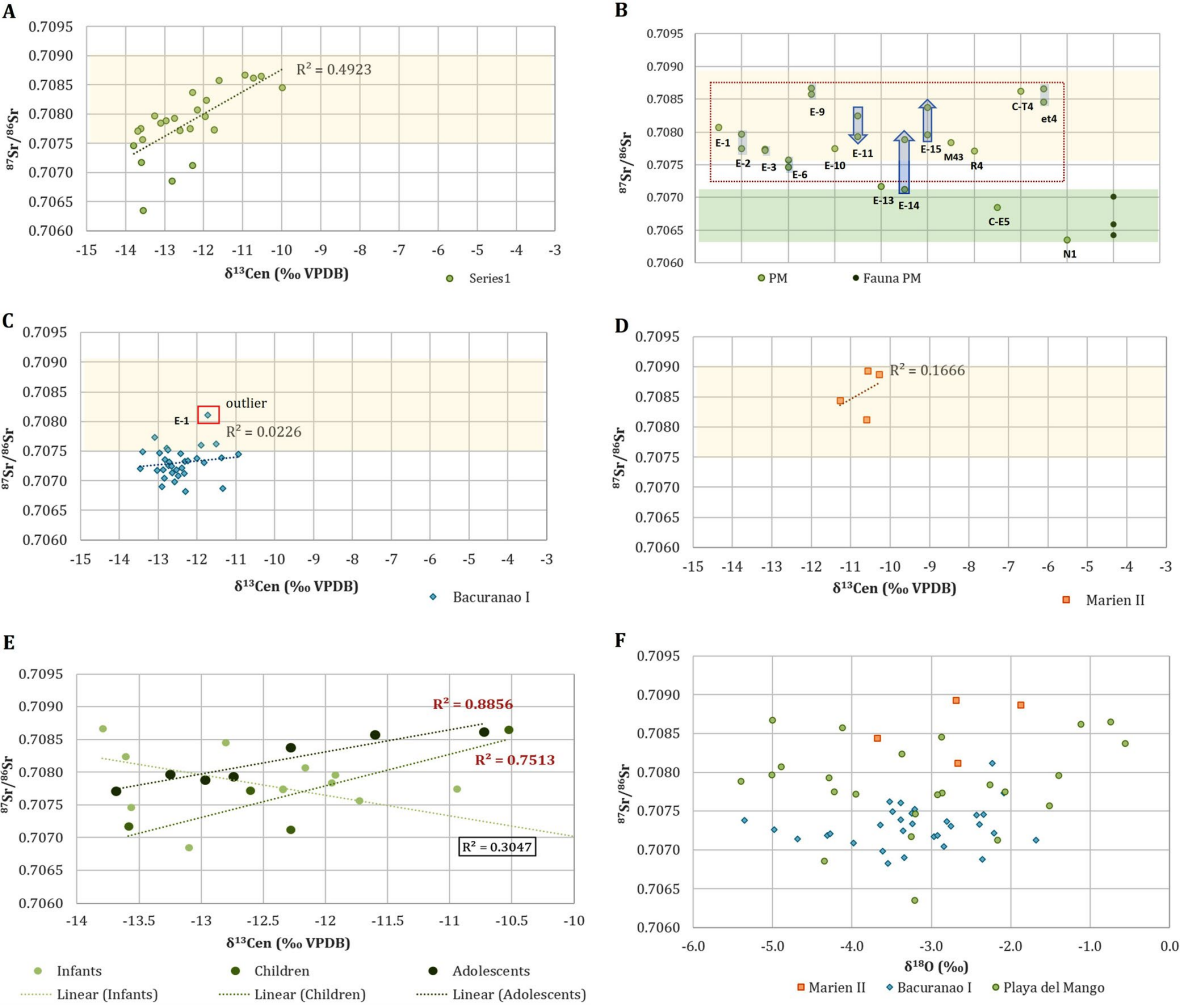
^87^Sr/^86^Sr and *δ* ^13^C_en_ values of individuals from Playa del Mango (A and B), Bacuranao I (C) and Marien II (D). Yellow and green shadowed areas represent the range of bioavailable ^87^Sr/^86^Sr as estimated from the bedrock and local hutias, respectively. Arrows in (B) show individuals for which tissues formed at different ages were sampled for ^87^Sr/^86^Sr analysis (run from earlier to later developing tooth). (E) ^87^Sr/^86^Sr and δ^13^C_en_ values of infants, children and adolescents from Playa del Mango; (F) ^87^Sr/^86^Sr and δ^18^O_en_ values of individuals from Marien II, Bacuranao I and Playa del Mango.

Hutias collected at the archaeological site of PM had an average ^87^Sr/^86^Sr of 0.70668 ± 0.00030 (Table 3). For human samples, ^87^Sr/^86^Sr ratios ranged between 0.70635 and 0.70867 (mean 0.70785 ± 0.00058, Figure 4A). No significant variations were found between tissues formed at different ages (*F* = 1.38, df = 2, *p* = 0.28). Only four individuals from PM (C‐E‐5, N1, E‐13, and E‐14) had ^87^Sr/^86^Sr values consistent with the bioavailable range of the site (Table 3, Figure 4B). Most individuals revealed similar ^87^Sr/^86^Sr values in tissues formed at different ages (variations ≤ 0.0002) while E‐11 (∆_M3−M1_ = 0.00031), E‐15 (∆_M3−M1_ = 0.00042), and E‐14 (∆_M3−M2_ = 0.00076) showed changes in their ^87^Sr/^86^Sr through life (Table 3, see arrows in Figure 4B).

**TABLE 3:** ^87^Sr/^86^Sr, *δ* ^13^C_en_, and *δ* ^18^O values of teeth from Playa del Mango (PM), Bacuranao I (CI), and Marien II (MII) individuals and hutia samples from PM.

ID	Teeth	Age at crown formation	^87^Sr/^86^Sr	*δ* ^13^C_en_ (‰)	*δ* ^18^O (‰)
PM_E‐1	M1	Infancy	0.70807	−12.2	−4.9
PM_E‐2	M1	Infancy	0.70775	−12.3	−4.2
	M3	Adolescence	0.70797	−13.3	−5.0
	∆_M3−M1_		0.00022	−1.0	
PM_E‐3	M1	Infancy	0.70774	−11.7	−2.9
	M2	Childhood	0.70772	−12.6	−4.9
	∆_M2−M1_		−0.00001	−0.9	
PM_E‐6	m2	Infancy	0.70757	−13.6	−1.5
	M1	Infancy	0.70747	−13.8	−3.2
	∆_M1−m2_		−0.00011	−0.2	
PM_E‐9	M1	Infancy	0.70867	−10.9	−5.0
	M3	Adolescence	0.70858	−11.6	−4.1
	∆_M3−M1_		−0.00009	−0.7	
PM_E‐10	M1	Infancy	0.70775	−13.6	−2.1
PM_E‐11	M1	Infancy	0.70824	−11.9	−3.4
	M3	Adolescence	0.70793	−12.7	−4.3
	∆_M3−M1_		−0.00031	−0.8	
PM_E‐13	M2	Childhood	0.70717	−13.6	−3.3
PM_E‐14	M2	Childhood	0.70712	−12.3	−2.2
	M3	Adolescence	0.70788	−13.0	−5.4
	∆_M3−M2_		0.00076	−0.7	
PM_E‐15	M1	Infancy	0.70796	−12.0	−1.4
	M3	Adolescence	0.70837	−12.3	−0.6
	∆_M3−M1_		0.00042	−0.3	
PM_M43	M1	Infancy	0.70784	−13.1	−2.3
PM_R4	M3	Adolescence	0.70771	−13.7	−2.9
PM_C‐E5	M1	Infancy	0.70685	−12.8	−4.4
PM_C‐T4	M3	Adolescence	0.70862	−10.7	−1.1
PM_eT4	P1	Childhood	0.70865	−10.5	−0.7
	M1	Infancy	0.70846	−10.0	−2.9
	∆_P1−M1_		0.00020	−0.5	
PM_N1	M1	Infancy	0.70635	−13.5	−3.2
Infant tissues					
Ave ± SD	*N* = 12 (11 individuals)	0.70775 ± 0.00062		−12.4 ± 1.5	−3.2 ± 1.2
Max/Min		0.708670/0.000266		0.6/−13.8	1.00/−5.0
Child tissues					
Ave ± SD	*N* = 5 (5 individuals)	0.70767 ± 0.00071		−12.2 ± 1.3	−2.5 ± 1.4
Max/Min		0.708654/0.70712		−10.5/−13.6	−0.7/−4.0
Adolescent tissues					
Ave ± SD	*N* = 7 (7 individuals)	0.70815 ± 0.00037		−12.5 ± 1.0	−3.4 ± 1.9
Max/Min		0.708621/0.707709		−10.7 ± −13.7	−0.6 ± −5.4
Total sample					
Ave ± SD	*N* = 24 (16 individuals)	0.70785 ± 0.00058		−12.4 ± 1.1	−3.1 ± 1.4
Max/Min		0.708670/0.706353		−10.0/−13.8	−0.7/−5.4
CI_AdultoB E‐1	C	Childhood	0.707362	−12.8	−2.8
CI_E‐1C	dc	Infancy	0.707458	−12.4	−2.2
	M1	Infancy	0.707470	−13.0	−2.3
	∆_M1−dc_		0.00001	−0.55	
CI_E‐1D	dm2	Infancy	0.707493	−13.4	−3.3
CI_E‐6	dm2	Infancy	0.707259	−12.7	−3.5
CI_E‐21B	dm2	Infancy	0.707620	−11.5	−5.0
CI_E‐21C	dc	Infancy	0.707331	−12.3	−3.5
CI_E‐21E	M1	Infancy	0.707042	−12.8	−2.4
CI_E‐21F	M1	Infancy	0.707173	−13.0	−2.8
CI_E‐28	dm2	Infancy	0.707386	−11.4	−3.0
CI_E‐29A	dc	Infancy	0.707377	−12.0	−3.4
CI_E‐30A	dm2	Infancy	0.707451	−10.9	−5.4
CI_E‐31	dm1	Infancy	0.707604	−11.9	−2.4
CI_E‐34A	dm1	Infancy	0.707126	−12.3	−3.4
CI_E‐35A	dm2	Infancy	0.707139	−12.6	−1.7
CI_E‐36A	dm1	Infancy	0.707546	−12.8	−4.7
CI_E‐39	M1	Infancy	0.707086	−12.5	−6.2
CI_E‐40A	dm2	Infancy	0.706875	−11.3	−4.0
CI_E‐49A	dm2	Infancy	0.707182	−12.9	−2.4
CI_E‐51A	dm2	Infancy	0.707523	−12.8	−2.9
CI_I22	M1	Infancy	0.707303	−11.8	−3.2
CI_I23	M1	Infancy	0.707317	−12.7	−2.8
CI_I25	M1	Infancy	0.706979	−12.6	−3.7
CI_I26	M1	Infancy	0.706903	−12.9	−3.6
CI_I27	M1	Infancy	0.707185	−12.5	−3.3
CI_I28	M1	Infancy	0.706822	−12.3	−4.3
CI_I29	M1	Infancy	0.707245	−12.7	−3.6
CI_I31	M1	Infancy	0.707214	−12.4	−3.4
CI_I41	M1	Infancy	0.707337	−12.2	−2.2
CI_I40	M1	Infancy	0.707206	−13.5	−3.2
CI_I43	M1	Infancy	0.707733	−13.1	−4.3
CI_E‐1	dm1	Infancy	0.708114	−11.7	−2.1
Ave ± SD	*N* = 32 (31 individuals)		0.70731 ± 0.00027	−12.4 ± 0.6	−3.4 ± 1.0
Max/Min			0.70811/0.70682	−10.9/−13.5	−1.7/−6.2
MII‐E1	M1	Infancy	0.708864	−10.3	−1.9
MII‐E2	M1	Infancy	0.708928	−10.6	−2.7
MII‐E18	M1	Infancy	0.708439	−11.3	−3.7
MII‐E21	M1	Infancy	0.708115	−10.6	−2.7
Ave ± SD	*N* = 4 (4 individuals)		0.70859 ± 0.00038	−10.7 ± 0.4	−2.7 ± 0.7
Max/Min			0.70893/0.70812	−10.3/−11.3	−1.9/−3.7
PM_hutia 1	I		0.70702		
PM_hutia 2	I		0.70642		
PM_hutia 3	I		0.70659		
Ave ± SD	*N* = 3 (3 hutias)		0.70668 ± 0.00030		
Max/Min			0.70702/0.70642		

*Note: N*: number of independent individuals.

Abbreviations: C: canine, I: incisor, P: premolar, M1, M2, M3: permanent first, second and third molars.

Individuals from PM had *δ* ^13^C_en_ values between −13.8‰ and − 10.0‰ (mean −12.4‰ ± 1.8‰) (Figure 4A, Table 3). The individuals within the local bioavailable ^87^Sr/^86^Sr range of the site had more restricted *δ* ^13^C_en_ values (from −13.6‰ to −12.3‰). Correlation between ^87^Sr/^86^Sr and *δ* ^13^C_en_ was 49% for the total sample (Figure 4A). However, tissues formed during childhood and adolescence showed a higher correlation between the variables (*R* ^2^ = 0.89 and *R* ^2^ = 0.75, respectively) than infant tissues (*R* ^2^ = 0.31) (Figure 4E). When individuals with ^87^Sr/^86^Sr ratios below 0.7072 were removed, there was a 70% correlation between the variables.

In addition, *δ* ^18^O_en_ values were more variable among individuals from PM (mean −3.1‰ ± 1.4‰, range −5.4‰ to −0.6‰) compared with those from either CI (mean −3.3‰ ± 1.0‰, range −6.2‰ to −1.7‰) or MII (mean −2.7‰ ± 0.7‰, range −3.7‰ to −1.9‰) (Table 3, Figure 4F). The individuals from PM who had ^87^Sr/^86^Sr values within the estimated range of local bioavailability (C‐E‐5, N1, E‐13, and E‐14) showed *δ* ^18^O_en_ values within a more restricted range than others from the site (Table 3, Figure 4F). No differences were found among tissues formed during infancy, childhood, or adolescence (*F* = 0.45, df = 2, *p* = 0.66).

#### 3.1.2 Bacuranao I Site

**FIGURE 5:**
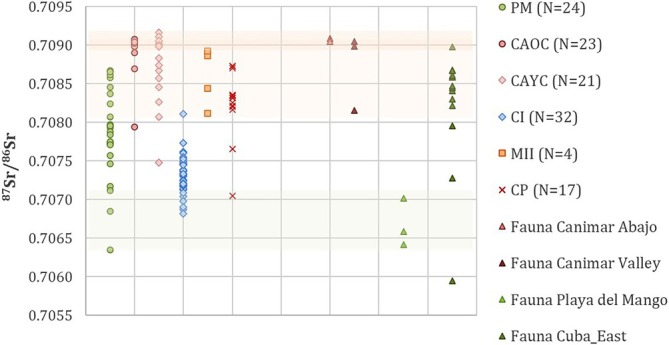
^87^Sr/^86^Sr ratios of individuals from Playa del Mango (PM), Bacuranao I (CI), Marien II (MII) and published values from Canímar Abajo (CAOC: Older cemetery; CAYC: Younger cemetery) and Cueva del Perico I (CP). Orange and green shadowed areas represent the expected ^87^Sr/^86^Sr range for the three regions examined and the range of bioavailable ^87^Sr/^86^Sr at PM site, respectively.

Individuals from the funerary site of CI had ^87^Sr/^86^Sr ratios that ranged between 0.70682 and 0.70811 (mean 0.70731 ± 0.00027) with the highest value identified as an outlier (*Z*‐score = 3.03) (Figure 4C). Most individuals had ^87^Sr/^86^Sr ratios lower than the values expected for the lithological unit in which the site is located (Figure 5). The *δ* ^13^C_en_ values ranged between −13.5‰ and −10.9‰ (mean −12.4‰ ± 0.6‰). The correlation between ^87^Sr/^86^Sr and *δ* ^13^C_en_ was very low (*R* ^2^ = 0.02) (Figure 4C). The *δ* ^18^O_en_ values ranged between −6.2‰ and −1.7‰ (mean −3.4‰ ± 1.0‰) (Figure 4F).

#### 3.1.3 Marien II Site

The ^87^Sr/^86^Sr ratios of individuals from MII ranged between 0.70812 and 0.70893 (mean 0.70859 ± 0.00038), which fell within the expected geoavailable ^87^Sr/^86^Sr range at the site (Figure 4D). In the case of *δ* ^13^C_en_, the values were between −11.3‰ and −10.3‰ (mean −10.7‰ ± 0.4‰). The correlation between ^87^Sr/^86^Sr and *δ* ^13^C_en_ was very low (*R* ^2^ = 0.16). Values of *δ* ^18^O_en_ ranged between −3.7‰ and −1.9‰ (mean −2.7‰ ± 0.7‰) (Figure 4F).

### 3.2 Variations in Diet

Statistically significant variations between sites were found for *δ* ^13^C_diet_ (*F* = 3.13, df = 6, *p* = 0.01), *δ* ^13^C_energy_ (*F* = 3.13, df = 6, *p* = 0.01), and *δ* ^13^C_protein_ (*H* = 45.31, df = 6, *p* < 0.01) values (Figure S1). Post hoc tests showed that variations in *δ* ^13^C_diet_ and *δ* ^13^C_energy_ were due to the statistically significant differences between the Marien II (MII) and Cueva Calero (CC) sites (*δ* ^13^C_diet_: df = 2.92; *p* = 0.04; *δ* ^13^C_energy_: df = 3.09; *p* = 0.04) (Figure S1A,B). Variations in *δ* ^13^C_protein_ were due to statistically significant differences between individuals from the Canímar Abajo (CA) site and most of the other sites. The isotopic composition of the protein source of the diet of individuals from CA OC differed significantly from those from Guayabo Blanco (GB) (*p* = 0.002), CC (*p* = 0.004), MII (*p* = 0.019), and Cueva del Perico I (CP) (*p* = 0.003), while the mean values of CA (younger cemetery [YC]) samples differed significantly from Playa del Mango (PM) (*p* = 0.013), GB (*p* = 0.000), CC (*p* = 0.000), MII (*p* = 0.010), and CP (*p* < 0.001) samples (Figure S1C). Mean values from PM individuals were also significantly different from those from GB (*p* = 0.012) and CP (*p* = 0.020).

In addition, the estimated values of *δ* ^13^C_diet_, *δ* ^13^C_energy_, and *δ* ^13^C_protein_ showed considerable intra‐site variation among individuals from CC, CP, and, to a lesser extent, the CA YC (Table 4). Less variation was observed between individuals from the other sites. Individuals from GB showed low variation in *δ* ^13^C_diet_ and *δ* ^13^C_energy_ values, but *δ* ^13^C_protein_ was highly variable (Table 4).

**TABLE 4:** *δ* ^15^N, *δ* ^13^C_co_, *δ* ^13^C_ap_, values of bones from Playa del Mango (PM), Guayabo Blanco (GB), Cueva Florencio (Fl), Cueva Calero (CC), Canímar Abajo (CAOC: Old cemetery; CAYC: Young cemetery), Bacuranao I (CI), Marien II (MII), and Cueva del Perico I (CP) individuals.

ID	*δ* ^15^N (‰)	*δ* ^13^C_co_ (‰)	*δ* ^13^C_ap_ (‰)	*δ* ^13^C_diet_ (‰)	*δ* ^13^C_energy_ (‰)	*δ* ^13^C_protein_ (‰)
PM2_E‐3	9.5	−18.6	−10.8	−20.4	−20.3	−23.8
PM2_E‐4	8.9	−16.1	−11.1	−20.7	−20.6	−20.1
PM2_E‐7	9.2	−17.6	−11.1	−20.7	−20.6	−22.2
PM2_E‐9	8.4	−16.7	−12.5	−22.2	−22.2	−20.2
PM2_E‐10	9.5	−15.9	−9.6	−19.2	−19.0	−20.8
PM2_E‐11	8.9	−18.9	−13.2	−22.9	−22.9	−22.7
PM2_E‐12	9.6	−18.6	−11.8	−21.5	−21.4	−23.2
PM2_E‐13	8.6	−17.4	−12.4	−22.1	−22.0	−21.1
PM2_E‐14	9.0	−18.9	−12.2	−21.9	−21.8	−23.2
PM2_E‐15	9.2	−18.2	−11.2	−20.9	−20.7	−22.9
PM1_M46	9.3	−17.3	−9.9	−19.5	−19.3	−22.5
PM2_E‐19	9.4	−18.4	−12.4	−22.1	−22.0	−22.5
PM1_M46	9.0	−18.6	−11.3	−21.0	−20.8	−23.4
Ave ± SD	9.1 ± 0.4	−17.8 ± 1.0	−11.5 ± 1.1	−21.16 ± 1.1	−21.1 ± 1.2	−22.2 ± 1.2
Max/Min	9.7/8.4	−15.9/−18.9	−9.6/−13.2	−19.2/−22.9	−19.0/−22.9	−20.1/−23.8
GB F1560	11.6	−19.2	−11.5	−21.1	−21.0	−24.2
GB F‐1190	10.7	−26.2	−10.0	−19.6	−19.4	−34.5
GB F‐1189	11.1	−20.2	−10.7	−20.3	−20.2	−26.0
GB F‐1192	16.0	−22.5	−11.6	−21.2	−21.1	−28.6
GB F‐1561	13.9	−24.8	−11.5	−21.2	−21.1	−31.7
Ave ± SD	12.64 ± 2.25	−22.6 ± 2.9	−11.1 ± 0.7	−20.7 ± 0.7	−20.6 ± 0.8	−29.0 ± 4.2
Max/Min	16.02/10.71	−19.2/−26.2	−9.9/−11.6	−19.6/−21.2	−19.4/−21.1	−24.2/−34.5
CC_E‐1 IV A	12.3	−20.1	−14.4	−24.2	−24.2	−23.6
CC_E‐1 IV B	6.3	−18.1	−12.6	−22.3	−22.2	−22.0
CC _E‐IV	11.6	−28.7	−15.1	−24.9	−25.1	−34.9
CC_E‐ XIV UH II	11.8	−20.9	−14.3	−24.1	−24.1	−24.9
CC_ E‐5	12.2	−21.5	−11.5	−21.1	−21.0	−27.3
CC _E‐1	7.6	−20.3	−9.8	−19.4	−19.2	−26.6
CC_E‐17	6.5	−19.8	−13.0	−22.7	−22.7	−24.0
Ave ± SD	9.75 ± 2.80	−21.32 ± 3.42	−12.95 ± 1.86	−22.7 ± 1.9	−22.7 ± 2.0	−26.2 ± 4.3
Max/Min	12.31/6.29	−18.05/−28.69	−9.84/−15.14	−19.4/−24.9	−19.2/−25.1	−22.0/−34.9
Fl_1	10.3	−21.7	−11.5	−21.2	−21.1	−27.5
Fl_2	10.4	−22.6	−12.6	−22.3	−22.2	−28.2
Ave ± SD	10.4 ± 0.1	−22.2 ± 0.6	−12.0 ± 0.8	−21.7 ± 0.8	−21.6 ± 0.8	−27.9 ± 0.4
Max/Min	10.4/10.3	−21.7/−22.6	−11.5/−12.6	−21.2/−22.3	−21.1/−22.2	−27.5/−28.2
CA_E‐19a	8.8	−14.2	−12.2	−21.9	−21.8	−17.0
CA_E‐19b	8.7	−13.0	−11.7	−21.4	−21.3	−15.6
CA_E‐87	10.9	−14.3	−12.2	−21.9	−21.9	−17.1
Ave ± SD	9.5 ± 1.2	−13.8 ± 0.7	−12.0 ± 0.3	−21.7 ± 0.3	−21.7 ± 0.3	−16.5 ± 0.8
Max/Min	10.9/8.7	−13.0/−14.3	−11.7/−12.2	−21.4/−21.9	−21.3/−21.9	−15.6/−17.1
CA_E‐2	9.1	−17.6	−11.0	−20.6	−20.5	−22.3
CA_E‐4	11.4	−13.0	−12.6	−22.3	−22.2	−15.1
CA_E‐6	10.6	−17.0	−11.0	−20.6	−20.5	−21.5
CA_E‐7	12.1	−18.1	−14.7	−24.4	−24.5	−20.8
CA_E‐10	9.2	−14.1	−12.3	−22.0	−22.0	−16.7
CA_E‐68	12.6	−11.7	−10.6	−20.2	−20.0	−14.5
CA_E‐69	11.3	−14.3	−12.0	−21.7	−21.6	−17.2
CA_E‐70	11.4	−16.3	−12.9	−22.7	−22.6	−19.4
CA_E‐72	11.8	−14.2	−11.4	−21.1	−20.9	−17.4
CA_E‐74	10.9	−12.6	−12.4	−22.1	−22.0	−14.7
CA_E‐75	9.4	−14.3	−12.6	−22.3	−22.3	−16.8
CA_E‐77	9.2	−16.0	−14.3	−24.1	−24.2	−18.1
CA_E‐80	12.3	−11.7	−12.4	−22.1	−22.0	−13.4
CA_E‐82	12.8	−17.3	−12.2	−21.9	−21.8	−21.1
CA E‐83	8.0	−14.6	−14.3	−24.0	−24.1	−16.3
CA_E‐84	10.8	−18.8	−14.7	−24.5	−24.6	−21.7
CA_E‐90	12.4	−14.2	−11.8	−21.5	−21.4	−17.2
CA_E‐91	11.7	−11.4	−11.9	−21.6	−21.53	−13.28
Ave ± SD	11.0 ± 1.4	−14.8 ± 2.3	−12.5 ± 1.3	−22.2 ± 1.3	−22.2 ± 1.4	−17.6 ± 2.9
Max/Min	12.8/8.0	−11.4/−18.8	−10.6/−14.8	−20.2/−24.5	−20.0/−24.6	−13.3/−22.3
E‐10	12.6	−19.5	−11.0	−20.6	−20.5	−24.9
E‐15	13.0	−18.2	−9.8	−19.4	−19.2	−23.8
E‐21	11.3	−17.9	−9.7	−19.3	−19.1	−23.4
Ave ± SD	11.7 **±** 1.3	−18.6 **±** 0.7	−10.2 **±** 0.7	−19.8 **±** 0.8	−19.6 **±** 0.8	−24.0 **±** 0.8
Max/Min	13.0/10.0	−17.9/−19.5	−9.7/−11.0	−19.3/−20.6	−19.1/−20.5	−23.4/−24.9
CP_Adulto A #2524	10.5	−18.2	−9.7	−19.3	−19.1	−23.9
CP_E‐36	9.0	−22.5	−12.0	−21.7	−21.7	−28.3
CP_E‐6A	10.5	−18.9	−10.6	−20.2	−20.0	−24.2
CP_E‐60	11.0	−18.4	−8.4	−18.0	−17.7	−24.8
CP_E‐39	12.0	−20.0	−13.5	−23.3	−23.3	−24.0
CP_E‐26B	9.8	−19.2	−13.3	−23.0	−23.0	−23.2
CP_E‐61	9.3	−22.7	−11.9	−21.6	−21.5	−28.6
CP_E‐28	13.6	−25.9	−11.1	−20.7	−20.6	−33.5
Ave ± SD	10.7 ± 1.5	−20.7 ± 2.7	−11.3 ± 1.7	−21.0 ± 1.8	−20.9 ± 1.9	−26.3 ± 3.6
Max/Min	13.6/9.0	−18.2/−25.9	−8.4/−13.5	−18.0/−23.3	−17.7/−23.3	−23.2/−33.5

**FIGURE 6:**
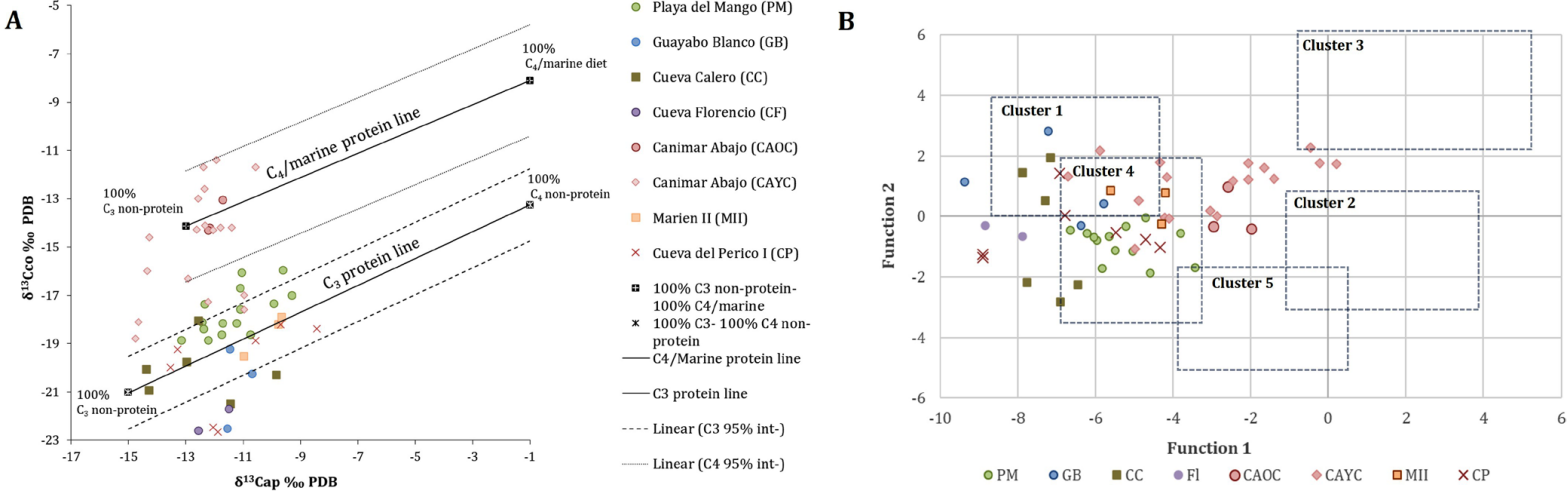
*δ* ^13^C_co_, *δ* ^13^C_ap_, and *δ* ^15^N_co_ values of Playa del Mango, Guayabo Blanco, Cueva Florencio, Cueva Calero, Canímar Abajo (older and younger cemeteries), Marien II, and Cueva del Perico I adults plotted against Froehle et al. (2010) (A) and Froehle et al. (2012) (B) models.

For most individuals, stable isotope data were distributed around the C_3_ protein line (and its 95% credibility range) as per the model of Froehle et al. (2010) and within Cluster 4 as per Froehle et al. (2012)'s model (Cluster 4: 70:30 C_3_/C_4_ diet with at least 65% C_3_ protein) (Figure 6A,B). In contrast, the individuals from the CA OC and most individuals from CA YC plotted more closely to the C_4_/ marine protein line (Figure 6A) in Froehle et al. (2010). Samples from the CA OC fell between Cluster 4 and 2 (Cluster 2: 30:70 C_4_/C_3_ diet; > 50% C_4_ protein). Samples from CA YC had greater dispersion, showing affinity with Cluster 4 and Cluster 1 (100% C_3_ diet/protein), the area in between Clusters 4 and 2, and the lower range of Cluster 3 (Cluster 3: 50:50 C_4_/C_3_ diet; marine protein). Individuals from Florencio (Fl), two individuals from CC, two from CP, and one from GB were below the 95% credibility interval of the C_3_ protein line (Figure 6A). Samples from those sites were grouped in Cluster 1 or around Cluster 1 and 4, as per Froehle et al. (2012) (Figure 6B).

**FIGURE 7:**
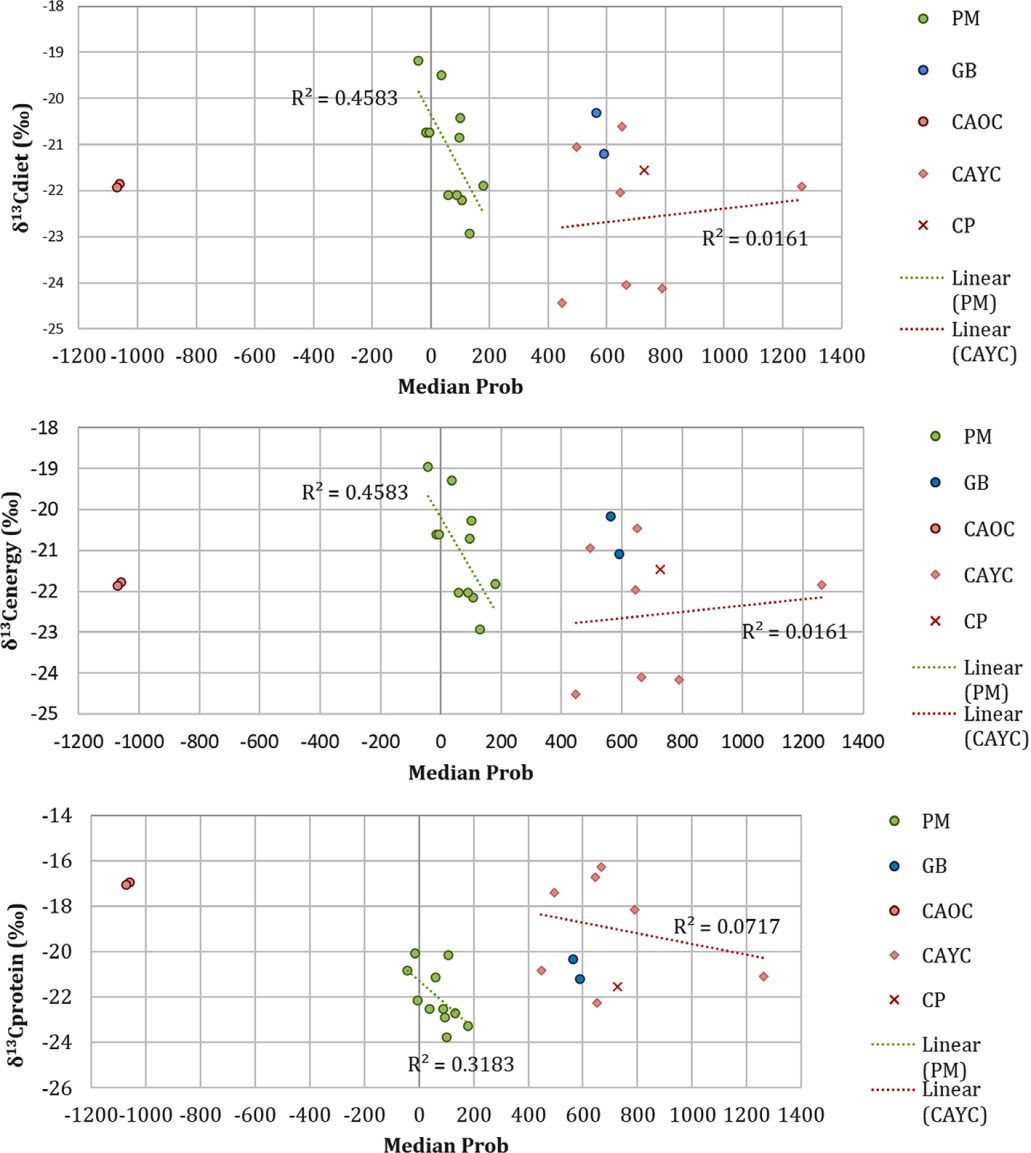
Changes in *δ* ^13^C_diet_, *δ* ^13^C_energy_, and *δ* ^13^C_protein_ over time. PM: Playa del Mango; GB: Guayabo Blanco; CAYC: Canímar Abajo younger cemetery; CAOC: Canímar Abajo older cemetery; CP: Cueva del Perico I.

The carbon isotopic composition of the diet and its energy and protein portions did not change significantly over time (Figure 7). In the case of PM, there was a weak tendency toward more depleted values in more recent individuals (*R* = 0.46). In the case of CA, variability increased among CA YC individuals in comparison to CA OC individuals (Figure 7).

## 4 Discussion

### 4.1 Diversity in Mobility Patterns

#### 4.1.1 The East of Cuba: Playa del Mango (cal. bce 116–241 ce)

Individuals from PM showed the greatest isotopic variability in both *δ* ^18^O_en_ and ^87^Sr/^86^Sr values. The site is located on the Quaternary deposits of the Cauto formation, where ^87^Sr/^86^Sr ratios are expected to be between 0.7075 and 0.7090 (Laffoon et al. 2012). Although most individuals had ^87^Sr/^86^Sr values consistent with that geoavailable range, four individuals showed ^87^Sr/^86^Sr values below 0.7072, consistent with the expected values of nearby areas where bedrocks are composed of older carbonate sediments from the Eocene‐Miocene (Figure 2). This would indicate that their teeth were possibly mineralized in areas underlain by volcanic bedrocks. Areas in the region where volcanic rocks are present are 65–100 km from PM.

In contrast with the expected geoavailable ^87^Sr/^86^Sr, the hutias from PM also showed low ^87^Sr/^86^Sr ratios (0.7067 ± 0.0003), which could suggest that lower values from human samples are in fact representative of the site area because of the contribution of different geological lithologies that drained into the delta (Bayon et al. 2021). It is also possible that the hutias were brought by humans from distant areas composed of volcanic rocks. This last scenario would suggest caution when using archaeological animals as a baseline for ^87^Sr/^86^Sr, as they could have been collected by humans from other lithological units.

Regardless of the local values, the population's ^87^Sr/^86^Sr isotopic diversity, including the variability observed between two teeth from the same individuals, suggests that they had high mobility within the Cauto Valley and surrounding areas. The high correlation found between *δ* ^13^C_en_ and ^87^Sr/^86^Sr ratios (70%) when the individuals with lower‐than‐expected ^87^Sr/^86^Sr ratios were removed (under 0.7072, possible locals) may indicate that this high mobility is linked to the collection or production of food sources between inland areas and areas closer to the coast within the Cauto Valley. A recent paleodietary study supported that the population of PM had a roughly 70:30 C_3_/C_4_ diet with high dependence on terrestrial animals (Chinique de Armas, González Herrera, et al. 2022). The strontium composition of omnivorous tissues is biased by strontium‐rich foods such as plants (Montgomery 2010). Different exogenous botanical resources such as tubers, maize, and beans have been identified in dental calculus (Chinique de Armas, González Herrera, et al. 2022) and in residues from artifacts (Rodríguez Suárez et al. 2020). It is likely that the PM population collected or farmed those plants in domestic gardens and different forest places within the Cauto region. It is possible that the low correlation between *δ* ^13^C_en_ and ^87^Sr/^86^Sr found in the first permanent molars is related to a different diet during the weaning process. It is also likely that ratios in these molars reflect the mother's values, which may suggest diverse provenance of females or high mobility not associated with food procurement. Dietary differences between adult females and males have also been reported at the site, which suggest differential distribution of activities between the sexes (Chinique de Armas, González, et al. 2022).

The pattern of high population mobility consistent with PM cultural tradition is supported by both the dispersion of archaeological evidence in the Cauto region (i.e., artifacts, faunal remains, and charcoals) and the type of raw materials used to make their artifacts. A wide circulation of volcanic rocks is apparent in the preparation of tools. This may denote systematic access to the areas where these raw materials are available. The closest rivers where raw material could have been collected are the Jicotea, Buey, and Yara rivers, which come from the Sierra Maestra and are located more than 15 km from the site. The deposits of the rocks recorded as part of their grinding‐maceration, and massive hammer technologies (Rodríguez Suarez et al. 2020) are from the mountains of the Santiago de Cuba (Southwest) and Holguín (Northeast) provinces. In the case of the rocks used in the carving of artifacts, the nearest resources are in the southwest of Holguín and toward the center‐south of Las Tunas (Instituto Cubano de Geodesia y Cartografía 1978).

#### 4.1.2 The West of Cuba: Bacuranao I (cal. 426–666 ce) and Marien II (cal. 474–597 ce)

The bedrocks around the Bacuranao I (CI) and Marien II (MII) sites are mostly composed of calcareous marls, limestones, conglomerates, and siliclastic limestones that are Pliocene‐Quaternary in age (Figure 3). The ^87^Sr/^86^Sr values of MII individuals were consistent with the expected values for those types of formations (0.7075 and 0.7090) (Laffoon et al. 2012), which indicate that the areas in which they obtained resources were restricted to Pliocene‐Quaternary lithological units. However, it is unlikely that all individuals sampled obtained their resources exclusively from coastal areas since both rain and soils receive significant contributions of marine‐derived strontium from sea spray, which has an ^87^Sr/^86^Sr ratio of ~0.7092 (Whipkey et al. 2000). Even though the burial site was only 500 m from the coast, it is likely that the village was more distant from the sea, and the range of mobility for food procurement covered more inland areas.

In the case of CI, most samples showed ^87^Sr/^86^Sr ratios lower than the expected geoavailable range, suggesting that those individuals were born or obtained foods from a place with lower isotopic values. A recent paleodietary study of the deciduous dentition and first permanent molars included in this study demonstrated that they were in the process of breastfeeding or weaning (Chinique de Armas, Mavridou, et al. 2022), suggesting that their ^87^Sr/^86^Sr ratios were representative of the mother's isotopic ratios. Volcanic rocks are not abundant in the site's vicinity, although small patches of gabbro and minor manifestations of ultramafic rocks (Chirino formation) can be found 11–15 km northeast of the site (Figure 3). The individual identified as an outlier (E‐1) had ^87^Sr/^86^Sr values consistent with the lithological unit of the site, which supports that a local origin. This infant was buried in a karstic sinkhole adjacent to the funerary space where the other nonadults were exhumed. A more detailed study of the ^87^Sr/^86^Sr bioavailability in the areas under study, including data from plants, rocks and local animals, will contribute to a better understanding of the mobility and provenance of individuals from Cuba.

### 4.2 Differences in Dietary Practices

In addition to the differences in mobility patterns, a considerable diversity in dietary traditions existed among early Cuban populations, and within some groups, in the estimated *δ* ^13^C_diet_, *δ* ^13^C_energy_, and *δ* ^13^C_protein_ values. The individuals from the older component of Canímar Abajo (CAOC) had a homogeneous diet that had a significantly different protein source from the later populations (Figure S1). This difference persisted between individuals from the younger cemetery of Canímar Abajo (CAYC) and the other groups from the west, suggesting that differences were connected to diversity of dietary traditions and not to geographic determinants. Further significant differences were found between individuals from PM and the other sites, except MII (believed to belong to the same archaeological tradition), in the protein portion of diet that have been estimated to be highly dependent on terrestrial sources (Chinique de Armas, González Herrera, et al. 2022).

The models of Froehle et al. (2010, 2012) show that early populations from Cuba fell within three general patterns. Some individuals from Guayabo Blanco (GB) and Cueva Calero (CC) clustered with populations with 100% C_3_ sources (terrestrial animals and C_3_ plants). Samples from PM, CC, CA YC, MII, and Cueva del Perico I (CP) fit within Cluster 4, which groups populations with 70:30 C_3_/C_4_ diets where at least 65% is C_3_ protein. This pattern has also been observed in later populations from the Greater Antilles associated with the Ceramic Age (Laffoon et al. 2016, 2020). This is consistent with a higher dependence on terrestrial resources and C_3_ plants, but with some contributions of C_4_/CAM plants. Maize, an exogenous cultigen, is the only C_4_ plant identified in the early sites of the Antilles (Pagán and Mickleburgh 2022) and maize starch grains have been found in the dental calculus of both CA and PM (Chinique de Armas et al. 2015; Chinique Armas, González Herrera, et al. 2022). It is possible that CAM plants such as agave, amaranths, and pineapple could also be responsible for the patterns observed.

Finally, individuals from CA were positioned between Clusters 4, 2, and 3 of Froehle et al. (2010)'s model, and showed high affinity for the C_4_ protein line (including the 95% credibility interval area, Figure 6). This finding supports previous results of high dependence on marine resources among CA populations (Chinique de Armas et al. 2015), which resembles the pattern observed in some populations from the Lesser Antilles (Laffoon et al. 2016, 2020). Some individuals from other sites, such as those from Florencio (Fl), were below the C_3_ protein 95% credibility line (Figure 6A), which may be explained by a mixed dietary pattern (Froehle et al. 2012). In general terms, the low correlation found between *δ* ^13^C_diet_, *δ* ^13^C_energy_, and *δ* ^13^C_protein_ and chronology suggests that the differences in diet were not associated with changes in environmental conditions or other natural factors over time but rather to different cultural traditions. The integration of new radiocarbon dates from human remains may help to clarify this hypothesis.

### 4.3 Diversity in Lifeways

Along with subsistence strategies, the level of residential mobility is a key variable to understanding people's adaptations and lifeways (Kent 2008). The results of this paper support that at least three distinct patterns of mobility, associated with different dietary signals, existed in early precolonial groups from Cuba. Between cal. bce 1320 and 807, the individuals from CA were likely linked to coastal areas around the CA site and had a more homogeneous mixed diet of mainly marine or riverine protein resources, but also terrestrial animals and C_3_/C_4_ plants (Chinique de Armas et al. 2025). A similar mixed pattern of protein consumption and low variability in ^87^Sr/^86^Sr ratios was reported at the Ortiz site in Puerto Rico (cal. bce 1780–800 [2*σ*]) (Pestle et al. 2023). The site of Ortiz resembles the CA OC in several aspects, including the selection of a rock shelter for funerary practices, the predominance of extended burial positions, and the apparent association of burials with charcoals and faunal remains. The low isotopic variability is also apparent among most individuals from Cueva del Perico I (CP) (Laffoon and Chinique de Armas Forthcoming). This is in sharp contrast with traditional narratives that described early Caribbean groups as foragers moving around different ecosystems (Rouse 1992). Unfortunately, no chronological data are available to support that this pattern is associated with the earlier component of the site, which may support that the earliest populations, who lived in a more humid environment (Haug et al. 2001), had access to preferred dietary sources within a more restricted area.

More variability in dietary practices and higher mobility was apparent among later groups. Between cal. bce 116 and 241 ce, the individuals from the “Banwaroid tradition” of Playa del Mango (PM) site had a high mobility pattern likely linked to the acquisition of food within the Cauto region and a mixed dietary pattern, whereby terrestrial animals and C_3_ plants had a high contribution to diet (Chinique de Armas, González Herrera, et al. 2022). The consistency of burial practices observed within the PM cemetery, and the fact that they occupied the site for centuries, may suggest that, in contrast with the effective mobility for food resources and raw materials, they had lower residential mobility.

From at least cal. 174 ce, a moderate pattern of mobility or different provenance, as well as a diversity in dietary traditions, was observed among groups in the west. The individuals from CA YC (cal. 332–1282 ce [2*σ*]) showed more isotopic variability than those from the older component of the site, as well as a higher number of nonlocals, which suggests the influx of individuals from other regions to the area and the use of the site by other populations within the Canímar Valley (Chinique de Armas et al. 2025). A more scattered pattern in the relationship between mobility and diet, as evidenced by the correlation between *δ* ^13^C_en_ and ^87^Sr/^86^Sr, was observed among individuals from CI and MII, although the conclusions on MII are constrained by the small sample size. Values of *δ* ^13^C_en_ are representative of total carbon (i.e., whole diet), including proteins, carbohydrates, and lipids (Krueger and Sullivan 1984; Schwarcz and Schoeninger 1991). The low correlation between the variables may be related to the acquisition of varied resources in a more isotopically restricted inland area.

Unfortunately, strontium isotopic values were not available for the other populations included in this study, which would help to further understand the potential diversity of mobility patterns among early groups or their relationship to their ecosystems or social organizations. However, the statistically significant differences observed in *δ* ^13^C_diet_, *δ* ^13^C_energy_, and *δ* ^13^C_protein_ between populations and the heterogeneity within groups support the notion that groups with different dietary traditions concurrently inhabited Cuba, at least from circa 174–761 ce (Figure 1B). In that sense, the higher intra‐site variability in groups such as CC, GB, or CP may be related to a more varied diet with access to less readily predictable available resources, or to the effect of the small sample sizes.

In addition to the differences previously described between CA and the other sites from western Cuba (CC, GB, and CP) (Chinique de Armas et al. 2016) and PM (Chinique de Armas, González Herrera, et al. 2022), there are relevant differences between GB and both CC (*δ* ^13^C_diet_ and *δ* ^13^C_energy_) and PM (*δ* ^13^C_protein_) individuals. However, the energy portion of the diets (correlated with whole diet) was highly variable within populations and more similar among groups than the protein sources. These differences in dietary practices were also observed between the only individual from the site of Maruca for whom stable isotope results are available and the four individuals studied at the Ortiz site in Puerto Rico (Pestle et al. 2023). Although the protein portion of diet seems to be influenced by biogeographic variables (Stokes 1998), with people from smaller and less biotically and geologically diverse islands showing a larger marine component in their protein diets, there is greater overall diversity among populations from Cuba than the rest of the Antilles combined (Chinique de Armas et al. 2016; Laffoon et al. 2016, 2020; Figure S2A,B).

The sites included in this study were in use as funerary places for a long period of time, making it difficult to draw definite conclusions in terms of populations in the absence of radiocarbon dates for all the individuals included in this study. However, because diet is linked to identity, the heterogeneity of lifeways as predicted by the different mobility patterns and dietary traditions showed in this work caution against using broad categories such as Archaic Age, *Preagroalfareros*, *Apropiadores* or *Ciboneyes* when referring to subsistence strategies or biology (e.g., Lalueza‐Fox et al. 2003; Fernandes et al. 2021). Understanding the particularities of these groups, and their population dynamics in time and space, may be necessary to shed light on the biological and cultural diversity of early Indigenous groups from the precolonial Antilles.

## Author Contributions

**Yadira Chinique de Armas:** conceptualization (lead), data curation (lead), formal analysis (lead), funding acquisition (lead), investigation (lead), methodology (equal), project administration (lead), resources (equal), software (lead), supervision (equal), validation (equal), visualization (lead), writing – original draft (lead), writing – review and editing (lead). **William Mark Buhay:** software (supporting), validation (equal), writing – review and editing (supporting). **Ulises Miguel González Herrera:** investigation (supporting), resources (equal), writing – review and editing (supporting). **Silvia Teresita Hernández Godoy:** data curation (equal), investigation (supporting), resources (supporting), writing – review and editing (supporting). **Jorge Fernando Garcell Domínguez:** investigation (supporting), resources (equal). **Luis Manuel Viera Sanfiel:** investigation (supporting), visualization (equal). **José Armando Caraballo Yera:** investigation (supporting), visualization (supporting). **Mirjana Roksandic:** funding acquisition (supporting), writing – review and editing (supporting). **Jason Laffoon:** data curation (equal), formal analysis (lead), funding acquisition (supporting), investigation (equal), methodology (equal), supervision (equal), validation (equal), writing – original draft (equal), writing – review and editing (supporting).

## Conflicts of Interest

The authors declare no conflicts of interest.

## Supporting information


Data S1.

## Data Availability

The data that supports the findings of this study are available in the main text and Supporting Information of this article.
