# Supplementary material for: Diversity of Lifeways in Early Antillean Societies: A Multi‐Isotope Approach
Source: Am J Biol Anthropol. 2025 Apr 16;186(4):e70039. doi: 10.1002/ajpa.70039 (PMC12003967; doi:10.1002/ajpa.70039)
Supplement: Supplementary file 1 — Data S1. [file AJPA-186-e70039-s001.pdf]

Appendix 1: Description of the sites

The archaeological site of **Playa del Mango** (PM) is in the Rio Cauto Basin, in the province of Granma. It is 3.5 km east of the lagoon system of Las Playas and 14 km north of the Gulf of Guacanayabo, in the southeast coast of Cuba. Two open site funerary areas have been identified: Mound 1 [cal AD 125 – 435 (2 σ)] and Mound 2 [cal. BC 116 and 241 AD (2σ)] (Chinique de Armas et al. 2020) with a minimum number of 21 individuals (Chinique de Armas et al., 2022a).

Guayabo Blanco´s mound is located in the south of Matanzas province. A minimum number of 12 individuals were exhumed from the site (see Cosculluela, 1965) with five stratigraphic levels: vegetal soil, shell, black soil, shell and limestone soil with the human remains (Cosculluela, 1965).

Cueva Florencio is a coastal site (1 and a half meters from the sea) nearby the Carbonera town. Secondary burials containing the skulls and long bones of four individuals, and a primary burial, were found at the site (Herrera & Rivero, 1954).

Cueva Calero is an inland funerary site in a cave located 2 km from the Camarioca River and, following the river, at 10 km from the north coast of Matanzas. A total of 54 skeletons in situ were excavated at the site (Martínez & Rives, 1990). In contrast, **Canímar Abajo** site in Matanzas has two funerary areas: the older cemetery [cal. BC 1320 – 807 (2σ)] and the younger cemetery [cal AD 332 – 1282 (2σ)] (Roksandic et al., 2015; Nägele et al., 2020), where remains of at least 220 individuals have been found (Roksandic et al. 2015). A carbon sample collected in the shell midden (UNAM-0715: cal. BC 5480 – 5380 (2σ) positioned the site as the oldest indigenous cemetery from the Greater Antilles (Roksandic et al. 2015; Napolitano et al., 2019).

The cave site of **Bacuranao I** (or Cueva del Infierno, CI) is 15 km inland from the north coast of Mayabeque in west Cuba. The site consisted of two funerary areas: Cemetery I

and II. Anthropological studies confirmed an MNI of 66 individuals including nine adults and 57 non-adults (Garcell, 2009).

The cave of **Marien** II (MII) is in the west of Bahia Honda and 500 m from the coast. The site consisted of two funerary areas: Cemetery I and II. Excavations at the site exhumed a total of 50 individuals that were classified as Ciboney Cayo Redondo type (La Rosa & Robaina, 1995).

Cueva del Perico I (CP) is located 1 km from both the Mani-Mani River and the north coast (Pino & Alonso 1973). Human remains from a minimum number of 162 individuals were excavated from the site (Travieso, García & Rivero, 1998).

Permits to study Playa del Mango were granted to Dr. Ulises M. González Herrera by the above-mentioned institution. Permits to study the samples from Bacuranao I and Marien II were granted to Jorge Garcell Domínguez. Permits to study the samples from Canímar Abajo were given to Dr. Silvia T. Hernández Godoy. Samples from Guayabo Blanco, Florencio, Cueva Calero, and Cueva del Perico I had been collected in 2010 under a project between the University of Havana and the University of Winnipeg. All human remains included in this study were treated with dignity and respect.

Appendix 2: Mean comparison Archaic Age sites Cuba

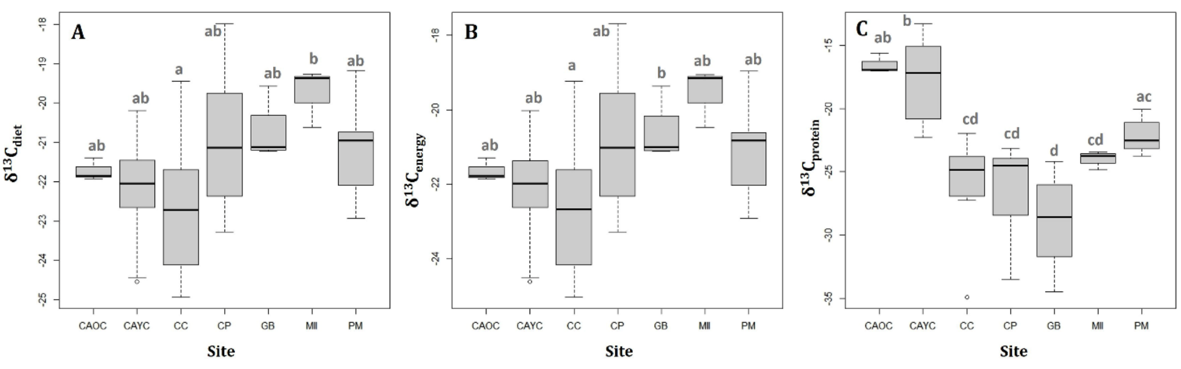

Figure S1. $\delta^{13}C_{diet}$, $\delta^{13}C_{energy}$ and $\delta^{13}C_{protein}$ mean comparisons for Playa del Mango (PM), Guayabo Blanco (GB), Cueva Florencio (Fl), Cueva Calero (CC), Canímar Abajo (older

and younger cemeteries), Marien II (MII) and Cueva del Perico I (CP) individuals. Different letters mean statistically significant differences.

Appendix 3: Isotopes values of Caribbean sites

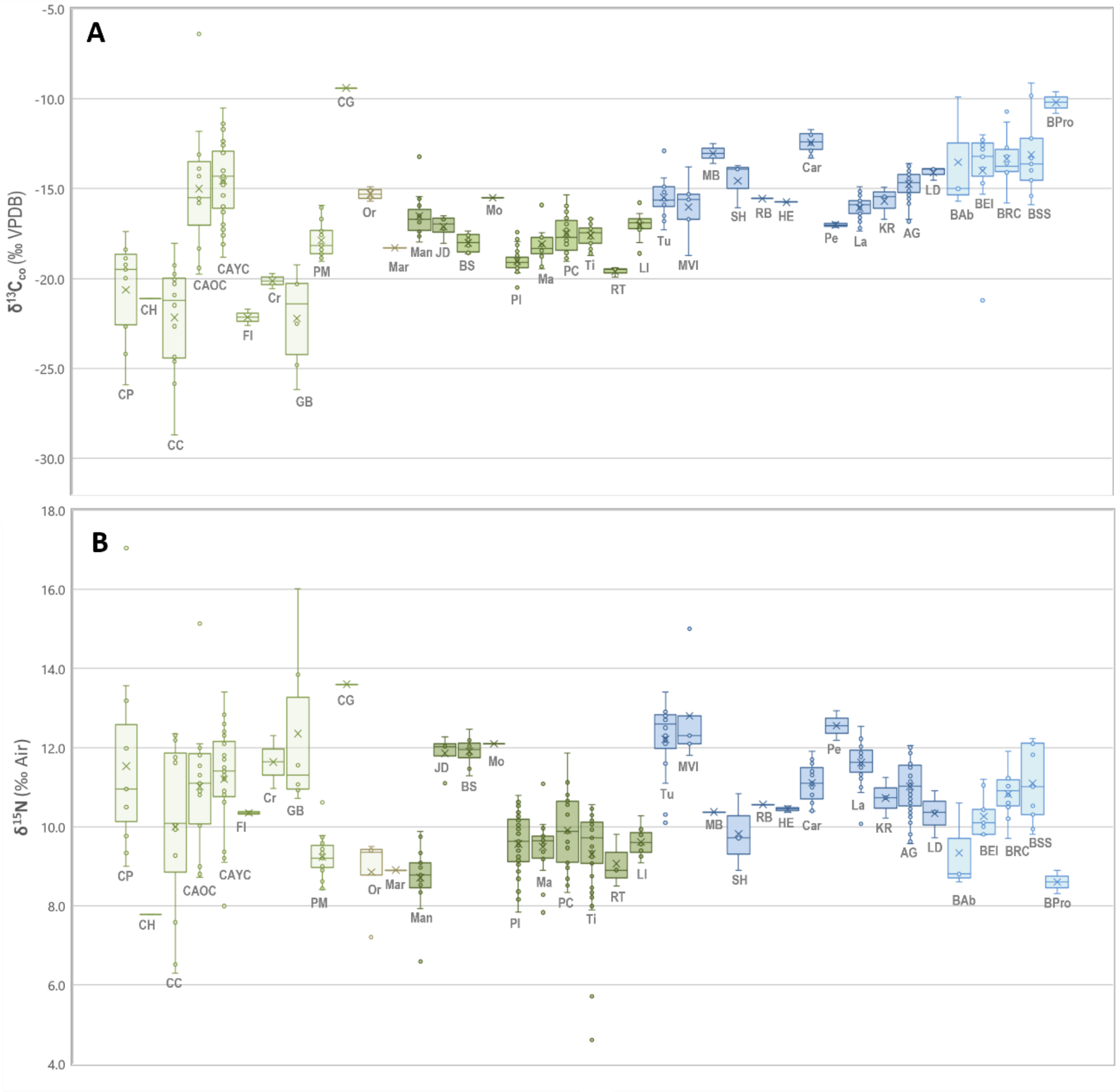

Figure S2. Carbon (A) and Nitrogen (B) isotope values of Caribbean populations. CP: Cueva del Perico I, CH: Cueva de los Hornos, CC: Cueva Calero, CAOC: Canímar Abajo, Old Cemetery, CAYC: Canímar Abajo Young cemetery, Fl: Florencio, Cr: Cueva Cristales, GB: Guayabo Blanco, PM: Playa del Mango, CG: Cueva de La Guinea (Cuba) (Chinique de Armas et al., 2015, 2016, 2022), Or: Ortiz, Mar: Maruca (Puerto Rico) (Pestle et al., 2023), Man: Manigat (Haití) (Stokes, 1998), JD: Juan Dolio, BS: Boca del Soco I (Dominican Republic) (Stokes, 1998), PI: Paso del Indio, Ma: Maisabel, PC: Punta

Candelero, Ti: Tibes, RT: Rio Tanama, LI: Los Indios (Puerto Rico) (Stokes, 1998; Pestle 2010; Pestle and Colvard, 2012), Tutu (Tutu) (Norr, 2002; Laffoon and Hoogland, 2012), MVI: Multiple Virgin Islands (Virgin Islands), MB: Maunday's Bay, SH: Sandy Hill, RB: Rendezvous Bay (Anguila) (Stokes, 1998), HE: Hope Estate (St. Martin) (Stokes, 1998), Car: Carriacou (Grenada) (Laffoon and Hoogland, 2012; Krigbaum et al. 2013), Pe:, La: Lavoutte (St. Lucia) (Laffoon et al., 2016), KR: Kelbey's Ridge (Saba) (Stokes, 1998), AG: Anse à la Gourde (Guadeloupe) (Stokes, 1998; Laffoon and De Vos, 2011), LD: , BAb: Abaco, BEI: Eleuthera, BRC: Rum Cay, BSS: San Salvador, BPro: Providenciales (San Salvador) in Bahamas (Keegan and DeNiro, 1988; Schulting et al., 2021). Light green: Early sites Greater Antilles; Dark green: Ceramic Age sites Greater Antilles; Dark blue: Ceramic Age sites Lesser Antilles, Light Blue: Bahamas.

References

Chinique de Armas, Y., Buhay, W. M., Rodríguez, R., Bestel, S, Smith, D., Mowat, S. D., & Roksandic, M. (2015). Starch analysis and isotopic evidence of consumption of cultigens among fisher–gatherers in Cuba: the archaeological site of Canímar Abajo, Matanzas. *Journal of Archaeological Science*, (58), 121-132. http://dx.doi.org/10.1016/j.jas.2015.03.003

Chinique de Armas, Y., González, U. M., Buhay, W. M., Yero, J. M, Viera, L. M., Burchell, M.,…& Roksandic, M. (2020). Chronology of the archaeological site of Playa del Mango, Rio Cauto, Granma, Cuba. *Radiocarbon*, 62 (5), 1-16. http://dx.doi.org/10.1017/RDC.2020.52

Chinique de Armas, Y., González, U. M., Reyes, I., Buhay, W. M., Skelton, S., Rodríguez, R.,…& Laffoon, J. (2022a). Multiproxy paleodietary reconstruction using stable isotopes and starch analysis: The case of the archaeological site of

Playa del Mango, Granma, Cuba. *Journal of Archaeological Science*, (46), 1 – 18. https://doi.org/doi:10.1016/j.jasrep.2022.103671

Chinique de Armas, Y., Roksandic M, Suárez, R. Smith, D. G., & Buhay, W. M. (2016). Isotopic evidence of variations in subsistence strategies and food consumption patterns among “Fisher-gatherer” populations of Western Cuba. In Roksandic, I. (Ed). *Cuban Archaeology in the Caribbean Context* (pp. 125-146). Gainsville: University Press of Florida.

Cosculluela, J. (1965). *Cuatro años en la Ciénaga de Zapata*. La Habana: Editorial E.C.A.G.

Garcell, J. F. (2009). *Arqueología en Bacuranao I. Nueva propuesta de categorías funerarias para las comunidades no ceramistas de Cuba*. La Habana: Editorial Unicornio.

Herrera, R., & Rivero, M. (1954). *La cueva funeraria de Carbonera*. La Habana: Editorial Sánchez.

Keegan, W. F., & DeNiro, M. J. (1988). Stable carbon-and nitrogen-isotope ratios of bone collagen used to study coral-reef and terrestrial components of prehistoric Bahamian diet. *American Antiquity*, 53 (2), 320-36. http://dx.doi.org/10.2307/281022

Krigbaum, J., Fitzpatrick, S. M., & Bankaitis, J. (2013). Human paleodiet at grand bay, Carriacou, Lesser Antilles. *The Journal of Island and Coastal Archaeology*, 8 (2), 210-27. http://dx.doi.org/10.1080/15564894.2012.756082

Laffoon, J. E., & Hoogland, M. L. (2012). Migration and mobility in the circum-Caribbean: integrating archaeology and isotopic analysis. In Kaiser, E., Burger, J., & Wolfram, S. (Eds), *Population Dynamics in Prehistory and Early History:*

New Approaches Using Stable Isotopes and Genetics (pp. 337-353). Berlin: De Gruyter.

Laffoon, J. E., Hoogland, M. L., Davies, G. R., & Hofman, C. L. (2016). Human dietary assessment in the Pre-colonial Lesser Antilles: New stable isotope evidence from Lavoutte, Saint Lucia. *Journal of Archaeological Science*, (5), 168-80. http://dx.doi.org/10.1016/j.jasrep.2015.11.020

Laffoon, J. E., & de Vos, B. (2011). Diverse origins, similar diets: an integrated isotopic perspective from Anse à la Gourde, Guadeloupe. In Hofman, C. L., & van Duijvenbode, A. (Eds), *Communities in Contact: Essays in Archaeology, Ethnohistory & Ethnography of the Amerindian Circum-Caribbean* (pp. 187-204). Leiden: *Sidestone Press.*

La Rosa, G., & Robaina, R. (1995). *Costumbres funerarias en aborígenes de Cuba*. La Habana: Multigraf.

Martínez, A., & Rives, A. (1990). Cueva Calero: recinto funerario aborigen de Cuba. *Revista Cubana de Ciencias Sociales*, 8 (24), 42-157.

Nägele, K., Posth, C., Orbegozo, M. I., Chinique de Armas, Y., Hernández Godoy, S. T., González, U. M.,…& Schroeder, J. (2020). Genomic insights into the early peopling of the Caribbean. *Science,* 369 (6502), 456-460. http://dx.doi.org/10.1126/science.aba8697

Napolitano, M. F., Dinapoli, R. J., Stone, J. H., Levin, M. J., Jew, N. P.,…& Fitzpatrick, S. M. (2019). Reevaluating human colonization of the Caribbean using chronometric hygiene and Bayesian modeling. *Science Advance*, 5 (12), eaar7806. https://doi.org/10.1126/sciadv.aar7806

Norr, L. (2002). Bone isotopic analysis and prehistoric diet at the Tutu site. In E. Righter (Ed.), *The Tutu Archaeological Village Site* (pp. 263-273). Routledge: New York.

Pestle, W. J. (2010). Diet and society in prehistoric Puerto Rico an isotopic approach. (Unpublished doctoral dissertation). University of Illinois, Chicago.

Pestle, W. J., & Colvard, M. (2012). Bone collagen preservation in the tropics: a case study from ancient Puerto Rico. *Journal of Archaeological Science*, 39 (7), 2079-2090. https://doi.org/10.1016/j.jas.2012.03.008.

Pestle, W. J., Pérez, E. M., & Koski-Karell, D. (2023). Reconsidering the lives of the earliest Puerto Ricans: Mortuary Archaeology and Bioarchaeology of the Ortiz site. *PLoS ONE*, 18 (4), e0284291. http://dx.doi.org/10.1371/journal.pone.0284291

Roksandic, M., Buhay, W. M., Chinique de Armas, Y., Rodríguez, R., Peros, M. C., Roksandic, I.,…& David, S. (2015). Radiocarbon and stratigraphic chronology of Canímar Abajo, Matanzas, Cuba. *Radiocarbon*, 57 (5), 755–763. https://doi.org/10.2458/azu_rc.57.18313

Schulting, R. J., Snoeck, C., Pouncett, J., Brock, F., Ramsey, C. B., Higham, T.,…& Ostapkowicz, J. (2021). Six centuries of adaptation to a challenging island environment: AMS 14C dating and stable isotopic analysis of pre-Columbian human remains from the Bahamian archipelago reveal dietary trends. *Quaternary Science Reviews*. http://dx.doi.org/10.1016/j.quascirev.2020.106780

Stokes, A. V. (1998). *A biogeographic survey of prehistoric human diet in the West Indies using stable isotopes*. (Unpublished doctoral dissertation). University of Florida, Florida.

Travieso, R., García, L., & Rivero, M. (1998). Estudio antropológico de los restos esqueletales aborígenes procedentes de la cueva del Infierno, San José de las Lajas, La Habana, Cuba. *Biología*, (12), 9-16.
